# Engineered Protein Nano-Compartments for Targeted Enzyme Localization

**DOI:** 10.1371/journal.pone.0033342

**Published:** 2012-03-12

**Authors:** Swati Choudhary, Maureen B. Quin, Mark A. Sanders, Ethan T. Johnson, Claudia Schmidt-Dannert

**Affiliations:** 1 Department of Biochemistry, Molecular Biology and Biophysics, University of Minnesota, St. Paul, Minnesota, United States of America; 2 University Imaging Centers, University of Minnesota, St. Paul, Minnesota, United States of America; Monash University, Australia

## Abstract

Compartmentalized co-localization of enzymes and their substrates represents an attractive approach for multi-enzymatic synthesis in engineered cells and biocatalysis. Sequestration of enzymes and substrates would greatly increase reaction efficiency while also protecting engineered host cells from potentially toxic reaction intermediates. Several bacteria form protein-based polyhedral microcompartments which sequester functionally related enzymes and regulate their access to substrates and other small metabolites. Such bacterial microcompartments may be engineered into protein-based nano-bioreactors, provided that they can be assembled in a non-native host cell, and that heterologous enzymes and substrates can be targeted into the engineered compartments. Here, we report that recombinant expression of *Salmonella enterica* ethanolamine utilization (*eut*) bacterial microcompartment shell proteins in *E. coli* results in the formation of polyhedral protein shells. Purified recombinant shells are morphologically similar to the native Eut microcompartments purified from *S. enterica*. Surprisingly, recombinant expression of only one of the shell proteins (EutS) is sufficient and necessary for creating properly delimited compartments. Co-expression with EutS also facilitates the encapsulation of EGFP fused with a putative Eut shell-targeting signal sequence. We also demonstrate the functional localization of a heterologous enzyme (β-galactosidase) targeted to the recombinant shells. Together our results provide proof-of-concept for the engineering of protein nano-compartments for biosynthesis and biocatalysis.

## Introduction

Engineering metabolic pathways into heterologous host cells to produce valuable chemical compounds and biofuels is a major goal of synthetic biology [1], [2]. Factors like diffusion limitation, alternate metabolic routes, accumulation of toxic reaction intermediates and inhibitory products, however, frequently reduce the efficiency of such engineered pathways. In nature, cells often circumvent these issues by co-localizing metabolic enzymes [3]. Co-localization can be achieved, for example, by tethering enzymes to structures such as protein scaffolds or lipid membranes [4], [5]. Drawing upon this approach, a synthetic protein scaffold was recently shown to dramatically increase flux through an engineered biosynthetic pathway [6]. Sequestration of enzymes into semi-permeable compartments or organelles is another strategy used by cells to spatially organize metabolic reactions. Unlike tethering, compartmentalization allows more stringent control over substrate and product transport to and from enzyme assemblies. It also protects the organism from harmful reaction intermediates. It is now known that several bacteria form proteinaceous shells that encapsulate functionally related enzymes. These are collectively referred to as bacterial microcompartments (BMCs) [7]. Recent advances in our understanding of BMC structure and function open up possibilities for engineering them into nano-bioreactors for biosynthesis and biocatalysis.

BMC-shell encoding genes are present in more than 400 sequenced bacterial genomes; and the encoded proteins are associated with enzymes involved in at least eight different metabolic pathways [7], [8], [9]. A subset of BMCs, called carboxysomes, plays an important role in CO_2_ fixation [10], [11]. Propanediol utilization (Pdu) BMCs catabolize 1,2-propanediol, and are found in *Salmonella*, *Citrobacter*, and some other bacteria [12], [13], [14]. *Salmonella* also forms BMCs during growth on the two-carbon substrate ethanolamine [7]. The membrane constituent phosphatidylethanolamine is hypothesized to serve as a major source of carbon, nitrogen and energy to enteric bacteria [7]. This view is supported by the attenuated behavior of *Salmonella* ethanolamine utilization (*eut*) loss-of-function mutants in the murine gut [15].

BMC shells have a viral capsid-like polyhedral structure with a diameter of 100–150 nm. While the shells of carboxysome are icosahedrons, those of Pdu and Eut BMCs appear to be semi-regular polyhedrons. Thin cell-section transmission electron micrographs of Eut BMCs indicate that Eut BMC shells have a rounder/less-sharp-edged morphology than either carboxysomes or Pdu BMCs [16], [17]. BMC shells are formed by thousands of copies of a few proteins belonging to the BMC-domain family (Pfam family: Pf00936). BMC-domain proteins have been crystallized as flat cyclic hexamers (or pseudohexamers), and are believed to form the edges and facets of the microcompartment [18]. The vertices of these shells are hypothesized to be capped by members of another BMC shell-associated protein family, some of which have been crystallized as pentamers (Pfam family: Pf03319) [7]. The multimeric shell protein structures have central pores of varying sizes and with different electrostatic properties. Crystallization of a few shell proteins with their central pores in either “open” or “closed” configuration suggests that they may function as gated transit points for cofactors and small metabolites [18].

**Figure 1:**
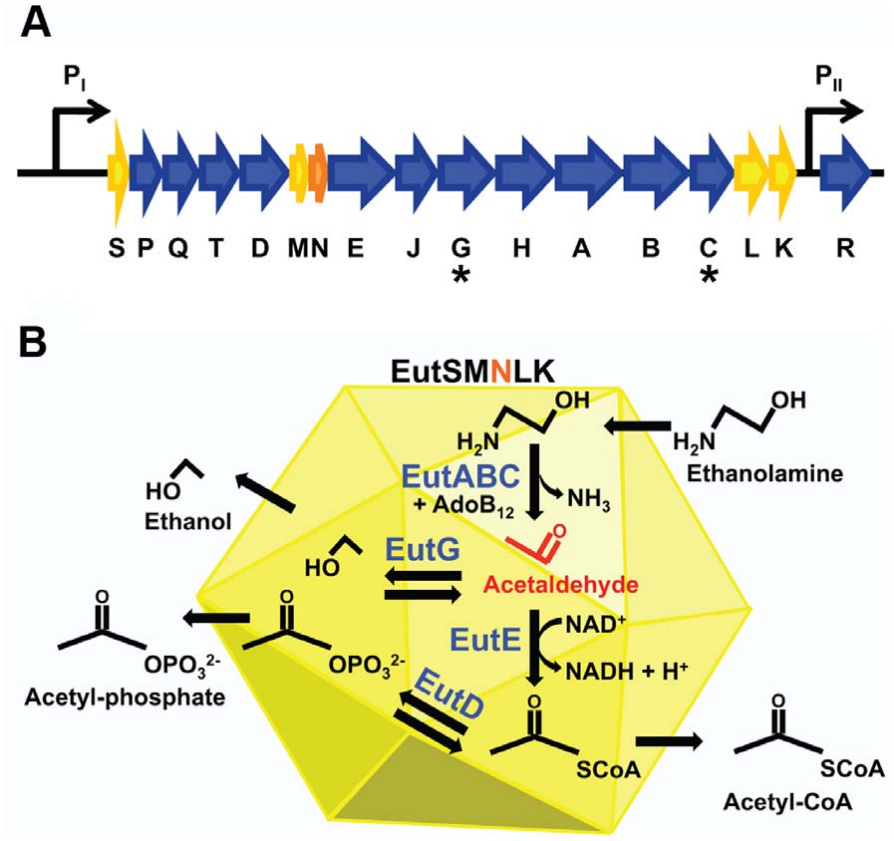
Coenzyme-B_12_-dependent ethanolamine utilization (*eut*) genes of *Salmonella enterica.* (**A**) *eut* operon in *S. enterica*. *eutS*, *eutM*, *eutN*, *eutL* and *eutK* encode BMC shell proteins that are proposed to form the Eut microcompartment (yellow and orange) [20]. Asterisks indicate genes that encode for enzymes with predicted N-terminal signal sequences that target them to the BMC interior [19]. Transcription is induced from the P_I_ promoter in the presence of both ethanolamine and vitamin B_12_, while the promoter P_II_ regulates weak constitutive expression of the transcription factor EutR [49]. (**B**) Model for catabolism of ethanolamine by the Eut BMC. Ethanolamine enters the microcompartment and is metabolized to ethanol, acetyl-phosphate and acetyl-CoA, which can enter the tricarboxylic acid cycle [7]. Eut BMC prevents dissipation of acetaldehyde, a volatile and toxic reaction intermediate (red) [21]. Enzymes assumed to reside in the BMC lumen include coenzyme-B_12_-dependent ethanolamine ammonia lyase (EAL, EutBC), EAL reactivase (EutA), alcohol dehydrogenase (EutG), aldehyde dehydrogenase (EutE), and phosphotransacetylase (EutD).

To demonstrate the feasibility of engineering heterologous protein-based microcompartments in *E. coli*, we chose the Eut BMC shell proteins from *Salmonella enterica* LT2. While the exact composition of *S. enterica* Eut BMCs is unknown, they are believed to be made up only five shell proteins; and putative signal sequences that target enzymes to the interior of Eut BMCs can be inferred from BMC-targeting sequences recently reported by Bobik and colleagues in experiments with native Pdu BMCs in *S. enterica* [19]. The Eut BMC shell genes are encoded on the 17-gene *eut* operon, which also encodes for enzymes involved in the degradation of ethanolamine (**Fig. 1**) [20]. Evidence suggests that *S. enterica* Eut BMCs prevent dissipation of the volatile reaction intermediate acetaldehyde, and protect the cell from aldehyde toxicity [8], [17], [21]. A homologous *eut* operon is also present in *E. coli*, but is disrupted by a transposon in several common laboratory strains. Although there have been some reports of *eut* operon induction and Eut BMC formation in a few *E. coli* strains, these were observed only under very specific growth conditions [16], [22]. Eut BMCs were not observed in our laboratory *E. coli* strains under either standard growth conditions or conditions reported to induce Eut BMC formation in *S. enterica*. To date, functional characterization of the *eut* operon has been conducted mainly using *S. enterica*; although crystal structures have recently been solved for putative Eut shell proteins cloned from *E. coli* [17], [21], [23], [24], [25], [26], [27], [28].

While there is interest in engineering bacterial microcompartments to create intracellular protein compartments for biotechnological applications, the interactions between individual BMC shell proteins and enzymes targeted for encapsulation have not been studied in any heterologous system [10], [19], [29]. Previously, an attempt was made to produce empty *Citrobacter* Pdu BMCs by overexpressing the Pdu shell proteins in *E. coli*; however, purification of intact microcompartments was not reported [30]. Here, we show that *E. coli* can form polyhedral compartments with recombinantly expressed *S. enterica* Eut shell proteins. Purified recombinant Eut shells appear to be morphologically similar to the native *S. enterica* Eut compartments. We demonstrate that an N-terminal signal sequence targets Enhanced Green Fluorescent Protein (EGFP) and β-galactosidase to the recombinant shells. The ability to sequester catalytically active β-galactosidase indicates that the recombinant compartments may be engineered to encapsulate multi-enzymatic reactions. We also report the surprising discovery that one of the BMC-domain proteins, EutS, is necessary and sufficient for the formation of shells *in vivo*, and for targeting of heterologous proteins to these structures; thereby offering a simple strategy for the engineering of protein-based nano-bioreactors.

## Results

### Expression of *S. enterica* Eut shell proteins in *E. coli*

The *eut* operon in *S. enterica* encodes for a Pfam03319 protein (EutN) and four BMC-domain proteins (EutS, EutM, EutL and EutK, Pfam00936) which are homologs of Eut and Pdu BMC shell-associated proteins from *E. coli* and *Citrobacter* [31]. In order to investigate the role of Eut shell proteins in heterologous shell assembly, we cloned the *S. enterica* Eut shell genes into our in-house BioBrick™ expression vector pUCBB (**Fig. S1**) [32]. In 3-dimensional (3-D) crystals, wild type EutS displays a hexameric structure with a bend of approximately 40°, while the EutS-G39V mutant forms flat symmetric hexamers [28]. We hypothesized that the unusual bent structure formed by wild type EutS is important for its role in BMC shell function. In order to test this hypothesis, the EutS-G39V mutant was also functionally characterized in this study. As shown in **Fig. S2**, EutS, EutS-G39V, EutM, and EutK were overexpressed as soluble proteins in two different *E. coli* strains, while the expression of recombinant EutN and EutL varied between the two strains. The soluble expression of EutL and EutK was also affected by the co-expression of other Eut shell proteins. EutS and EutM showed aberrant SDS-PAGE migration (“smearing”) typical of proteins that have hydrophobic peptide stretches [33].

### Formation of polyhedral microcompartments in *E. coli* co-expressing all five Eut shell proteins

**Figure 2:**
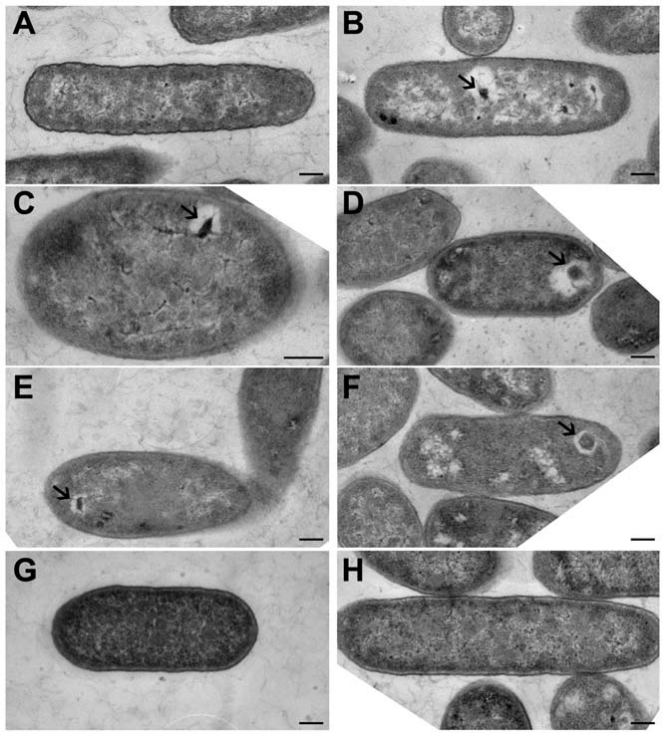
Formation of engineered protein shells by expression of *S. enterica* Eut shell proteins in *E. coli*. Transmission electron micrographs of thin sections of *S. enterica* and recombinant *E. coli*. (**A**) *S. enterica* grown on glycerol. (**B**) *S. enterica* grown on ethanolamine. (**C**) *E. coli* C2566 expressing recombinant EutSMNLK. (**D**) *E. coli* JM109 expressing recombinant EutSMNLK. (**E**) *E. coli* C2566 expressing recombinant EutS. (**F**) *E. coli* JM109 expressing recombinant EutS. (**G**) *E. coli* C2566 expressing recombinant EutMNLK. (**H**) *E. coli* JM109 expressing recombinant EutMNLK. Arrows indicate the location of recombinant BMCs. (Scale bar: 200 nm).

Our next goal was to ascertain if the recombinant Eut shell proteins formed clearly demarcated protein shells in *E. coli*. For this purpose, cultures of *E. coli* harboring all five *S. enterica* Eut shell genes were grown overnight at 30°C. This lower temperature was expected to reduce the expression of individual recombinant proteins, and supply sufficient time for assembly of the 3-D shell. The cells were fixed, sectioned, and their internal structures examined by transmission electron microscopy (TEM). Expression of all five Eut shell proteins in *E. coli* resulted in the creation of clearly discernible protein shells in most of the examined cells (**Table S1**). The observed recombinant protein shells bore a strong resemblance to the BMCs produced by *S. enterica* control cells growing on ethanolamine (**Fig. 2A–D**, additional images in **Fig. S3**). The recombinant compartments were 100–200 nm in diameter and within range of the dimensions previously reported for native Eut BMCs [7]. However, while *S. enterica* cells displayed several Eut BMCs distributed through-out the cell, the recombinant EutSMNLK structures were restricted to only one or two per *E. coli* cell. Their intracellular localization was such that they were situated off-center, and close to the poles of the cell. These recombinant microcompartments were observed in two different strains of *E. coli* (C2566 and JM109). The structures formed in JM109 cells were larger in size than those observed in C2566 cells, a result likely arising from differences in shell protein expression rates between the two strains. The EutSMNLK shells were enveloped by an electron-transparent region which was also observed around many of the native *S. enterica* Eut BMCs. The Eut protein compartments may therefore be enveloped by an unknown matrix which is removed by detergent wash, suggesting that it may be of a lipophilic nature.

### EutS alone is able to form protein shells in *E. coli*

Based on the crystal structures of Eut shell proteins, a model has been proposed for their assembly to form the microcompartment [27], [28]. According to this model, the edges and facets of the shell are formed by EutS and EutM respectively, while the central pore of the EutL pseudohexamer may facilitate gated transport into and out of the BMC. EutN is expected to ‘cap’ the vertexes of the icosahedral capsid, although unlike other Pfam03319 proteins which form pentamers, it crystallizes as a hexamer. The role of EutK in the Eut microcompartment assembly and function is at present unclear.

To explore the roles of *S. enterica* Eut shell proteins in the assembly of the Eut microcompartment, they were expressed in *E. coli* both individually and in different combinations. To our surprise, recombinant expression of EutS alone resulted in the formation of protein shells which were similar to those observed with EutSMNLK (**Fig. 2E–F**, additional images in **Fig. S3**). Engineered EutS protein shells appeared to be morphologically similar to the EutSMNLK compartments – they too were well-delimited and were surrounded by an electron-transparent region. One to two polyhedral bodies were observed per cell, and their appearance and localization matched that of compartments produced by EutSMNLK in *E. coli*. The ratio of 90 nm thin cell sections displaying recombinant compartments indicates that a majority of the *E. coli* cells expressed EutS compartments (**Table S1**). As shown in **Fig. 2G–H**, no defined structures were observed in cells co-expressing EutMNLK. These results indicate that engineering of *S. enterica* EutS is sufficient and necessary to form recombinant shells in *E. coli*.

Structures formed by over-expression of other Eut shell proteins are shown in **Fig. S3**. Recombinant expression of EutM alone created a thick axial filamentous structure which allowed cell division but interfered with separation. Similar fibers have been reported in *E. coli* expressing *Citrobacter freundii* PduAB, as well as the *S. enterica pduJ* deletion mutant [30], . Expression of EutK alone resulted in the formation of an electron-translucent region within the cell, while an electron-dense region was produced by co-expression of EutM and EutN. Comparable amorphous aggregates (albeit of a smaller size) are formed by *S. enterica pduBB'* loss-of-function mutants [34]. A small fraction of cells co-expressing EutLK displayed internal filaments similar to those observed in *E. coli* expressing *Citrobacter* PduABKN [30]. Apart from a few polar granules, *E. coli* expressing either EutN or EutL alone did not display clearly discernible structures.

### An N-terminal signal sequence targets EGFP to Eut shells

**Figure 3:**
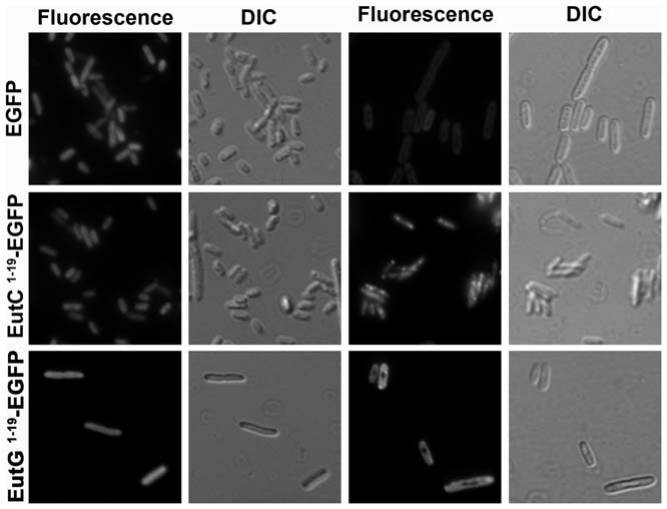
Distribution of EGFP bearing putative N-terminal Eut BMC-targeting signal sequences in *S. enterica*. *S. enterica* cells containing constructs for constitutive expression of EGFP, EutC^1–19^-EGFP or EutG^1–19^-EGFP were cultured with either glycerol or ethanolamine. Distribution of green fluorescence within the cells was observed by fluorescence microscopy. DIC images show the cell boundaries.

Recently, Fan *et al*. have demonstrated that an N-terminal signal sequence of PduP, a Pdu BMC-associated enzyme, targets heterologous proteins to the interior of native Pdu microcompartments in *S. enterica* [19]. Their sequence analysis also predicted N-terminal targeting sequences for other BMC-lumen associated proteins, including EutC and EutG (**Fig. 1**). In order to test if the predicted signal sequences (the first nineteen amino acids of EutC and EutG: EutC^1–19^ and EutG^1–19^, respectively) indeed function as Eut BMC-targeting sequences, we fused them to the N-terminus of EGFP; and expressed the fusion proteins in *S. enterica* grown in the presence of either glycerol or ethanolamine (to induce BMC formation). Bright fluorescent localization was observed in EutC^1–19^-EGFP expressing cells grown on ethanolamine, suggesting that the tagged EGFP was being targeted to the Eut BMCs (**Fig. 3**). We noticed that the fluorescent loci were not stationary but were moving around within cells, suggesting interaction of the Eut BMCs with the *S. enterica* cytoskeleton (**Video S1**).

No fluorescent localization was observed when EutC^1–19^-EGFP expressing cells were grown on glycerol, or with the EGFP control. Cells expressing EutG^1–19^-EGFP also failed to display punctate green fluorescence when grown on ethanolamine, indicating that fusion with the first nineteen amino acids of EutG is not sufficient to target heterologous proteins to the Eut BMC.

**Figure 4:**
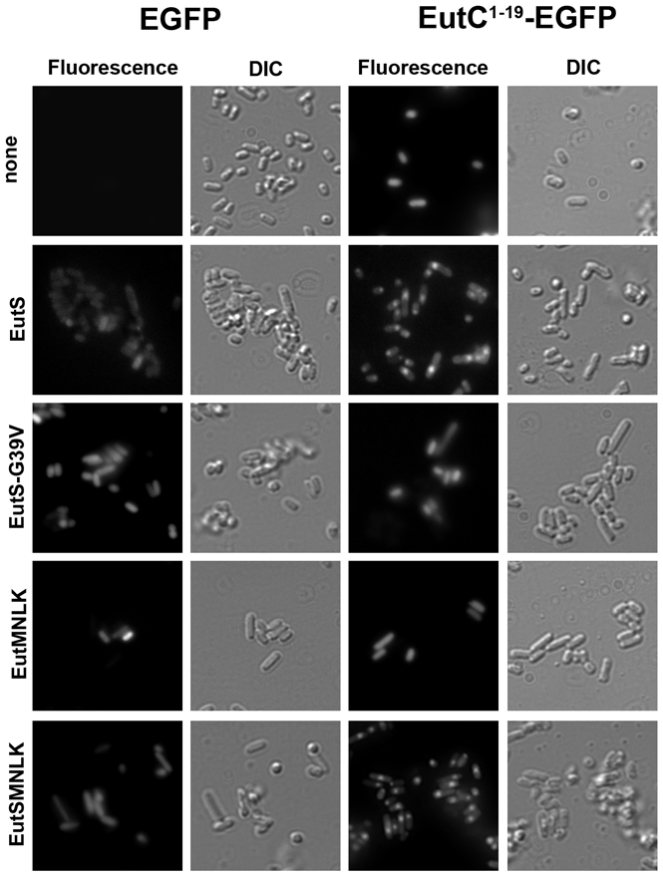
Localization of EutC^1–19^-EGFP in recombinant *E. coli* expressing *S. enterica* Eut shell proteins. Fluorescence microscopy images of *E. coli* C2566 cells co-expressing EGFP or EutC^1–19^-EGFP with EutS (wild type or the G39V mutant), EutMNLK or EutSMNLK. Cell boundaries are shown by the DIC images. (see **Table S2** for the quantification of EGFP localization in recombinant *E. coli*, and **Fig. S4** for the localization of EutC^1–19^-EGFP in the *E. coli* JM109 strain).

After establishing that the first nineteen amino acids of EutC functioned as a BMC-targeting sequence in *S. enterica*, we sought to explore whether this sequence also localized heterologous proteins to the recombinant Eut protein shells engineered in *E. coli*. Therefore, EutC^1–19^-EGFP was expressed in *E. coli* cells harboring the entire complement of Eut BMC shell proteins (EutSMNLK). Strong localized fluorescence was observed in 84% of the recombinant *E. coli* C2566 cells (**Fig. 4** **, Table S2**). Fluorescent green foci were also observed by co-expression of EutC^1–19^-EGFP and EutSMNLK in *E. coli* JM109 cells; which indicates that the effect is not strain-specific (**Fig. S4, Table S2**). The number and location of these fluorescent foci (one to two per cell, near the poles) are consistent with the capsid structures observed by TEM. Transmission electron micrographs of thin cell sections indicated that similar structures were formed by *E. coli* cells expressing recombinant EutSMNLK alone or in combination with EutC^1–19^-EGFP, supporting previous reports that formation of the bacterial microcompartment *in vivo* does not require scaffolding provided by a cargo protein (**Fig. S3**) [30], [35]. Unlike in *S. enterica*, fluorescent loci in *E. coli* cells co-expressing EutSMNLK with EutC^1–19^-EGFP appeared to be stationary.

### EutS is sufficient and necessary for targeting EutC^1–19^-EGFP to recombinant Eut shells

To investigate whether one or a combination of Eut shell protein(s) is required for EGFP localization in *E. coli*, we next co-expressed EutC^1–19^-EGFP with various Eut shell proteins. Remarkably, co-expression of only EutS and EutC^1–19^-EGFP resulted in the formation of fluorescent foci within 87% of the *E. coli* C2566 cells and 84% of the *E. coli* JM109 cells, while cells expressing other Eut shell protein combinations without wild type EutS did not display discrete fluorescent localizations (**Fig. 4** **, Fig. S4, Fig. S5, Table S2**). Our results therefore indicate that EutS is necessary and sufficient for targeting EutC^1–19^-EGFP to the engineered compartment.

### EutS and EutSMNLK shells are neither inclusion bodies nor are they enveloped by a hydrophobic matrix

The red fluorescent, lipophilic stain Nile Red was employed to examine the composition of the matrix surrounding the engineered proteins shells and to confirm that the shells were not inclusion bodies [36]. *E. coli* cells co-expressing EutC^1–19^-EGFP and Eut shell proteins were stained with Nile Red, reasoning that co-localization of red and green fluorescent foci would indicate that the observed shells are either enveloped by a hydrophobic layer or are made up of aggregated, mis-folded proteins (aka. inclusion bodies). As shown in **Fig. S6**, no such co-localization was observed, indicating that the engineered protein shells formed by EutS alone or EutSMNLK are not inclusion bodies nor are they surrounded by a lipophilic matrix. As a control, we stained with Nile Red *E. coli* cells co-expressing the cyanobacterial carotenoid cleavage dioxygenase NSC-1, which we have previously shown to form inclusion bodies [37], and EutC^1–19^-EGFP. As expected, clear Nile Red deposits were observed in *E. coli* expressing NSC1 inclusion bodies (**Fig. S7**). Furthermore, we observed dispersed green fluorescence, indicating that EutC^1–19^-EGFP is not targeted to the NSC1 inclusion bodies. Therefore, our results indicate that overexpressed EutS or EutSMNLK do not form inclusion bodies in *E. coli* nor are the shells enveloped by a hydrophobic layer. The composition of the matrix surrounding the shells (appearing as electro-transparent region after detergent wash) remains unclear.

The localization of EutC^1–19^-EGFP but not EGFP to either EutS or EutSMNLK protein shells also shows that the recombinant protein compartments are made of functional, properly folded proteins; and are not merely aggregates of mis-folded proteins. Further evidence for the protein shells not being inclusion bodies is provided by the absence of EutC^1–19^-EGFP puncta in *E. coli* cells co-expressing the EutS-G39V mutant (**Fig. 4** **, Fig. S4, Table S2**). Crystallographic studies have shown that this particular mutation causes a major change in the conformation adopted by EutS hexamers [28]. Our data links the altered 3-D crystal structure of EutS-G39V with an inability to sequester EutC^1–19^-EGFP, suggesting that the conformation adopted by properly folded EutS-WT *in vivo* is essential for targeting EutC^1–19^-EGFP to the engineered protein shell.

### Purification of Eut microcompartments

To further characterize Eut microcompartment formation in *E. coli*, we sought to purify the recombinant compartments. To the best of our knowledge, there have been no previous reports of successful purification of *S. enterica* Eut microcompartments. We therefore chose to first establish a protocol for purifying native Eut BMCs from *S. enterica* grown on ethanolamine. To assist in tracking fractions containing Eut BMCs during ultracentrifugation steps, we used *S. enterica* cells expressing EutC^1–19^-EGFP, anticipating that encapsulation of EutC^1–19^-EGFP by Eut BMCs would make them visible under UV light. Native Eut compartments were then isolated by modifying a previously established protocol reported for native *S. enterica* Pdu BMCs [13]. As a negative control we performed the same purification procedures with *E. coli* C2566 cells expressing only EutC^1–19^-EGFP.

**Figure 5:**
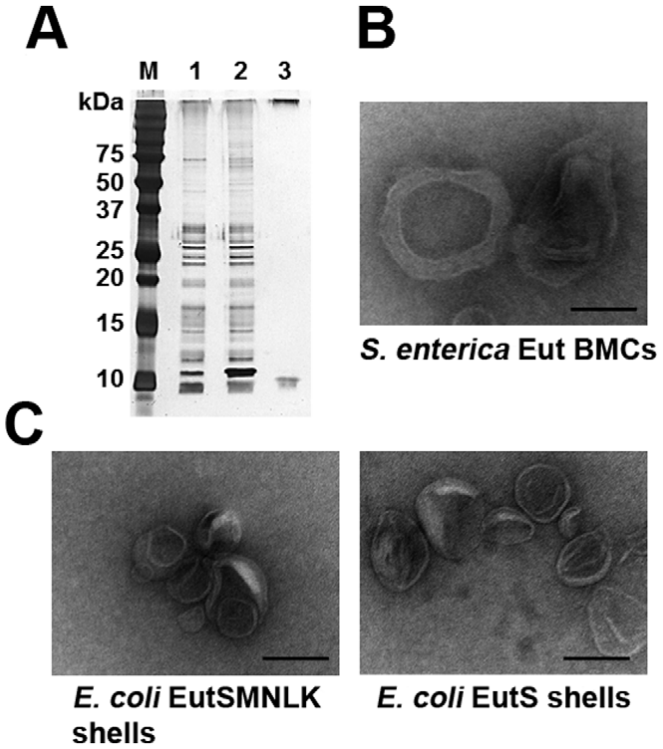
Purification of Eut compartments. (**A**) Silver stained SDS-PAGE gel showing purification of (lane 1) Eut BMCs from *S. enterica* cells harboring EutC^1–19^-EGFP, (lane 2) recombinant EutSMNLK BMCs, and (lane 3) recombinant EutS BMCs from *E. coli* C2566 cells co-expressing EutC^1–19^-EGFP. Calculated protein sizes are as follows: EutS (11.6 kDa), EutM (9.8 kDa), EutN (10.4 kDa), EutL (22.7 kDa), EutK (17.5 kDa), EutC^1–19^-EGFP (29.1 kDa). (**B**) Transmission electron micrographs of isolated native and recombinant Eut compartments. From left to right: Eut BMCs from *S. enterica*, EutSMNLK shells from *E. coli* C2566, EutS shells from *E. coli* C2566. (Scale bar: 100 nm).

A white band that was fluorescent under UV light after sucrose gradient ultracentrifugation of *S. enterica* cell lysate was collected for further analysis. The *E. coli* EutC^1–19^-EGFP control revealed a faint white band, but no fluorescent bands, in the sucrose gradient that was also collected. Isolated fractions were then analyzed by SDS-PAGE and TEM. SDS-PAGE analysis of the *Salmonella* sample (**Fig. 5A** **, lane 1**) showed a number of protein bands, including bands with molecular weights expected for recombinant Eut shell proteins and EGFP (**Fig. S2**). Aberrant protein migration (“smearing”) of low molecular weight bands occurred similar to what was previously seen with the expression of EutS and EutM in *E. coli*. SDS-PAGE analysis of the control fraction from *E. coli* EutC^1–19^-EGFP did not show any protein bands.

Negative stain TEM of the purified native Eut organelles revealed structures which were irregular in shape, with dimensions in the range of 100–150 nm (**Fig. 5B**). The purification was reproduced several times and negatively-stained structures of the same morphology were viewed on several different occasions, indicating that the structures isolated and viewed are the native Eut BMCs. The *E. coli* EutC^1–19^-EGFP control sample did not contain any structures that could be visualized by TEM, confirming that the visualized *Salmonella* compartments are Eut BMCs and not membrane vesicles.

As an additional control, native Pdu BMCs from *S. enterica* were also purified and visualized following the published method (**Fig. S8A**) [13]. In our hands, the morphology of the purified Pdu and Eut BMCs is very similar – the structures are clearly discernible but somewhat deflated, perhaps as the result of the purification procedure and/or the destructive nature (dehydration) of negative stain EM.

Subsequently, we applied the same purification procedure developed for native Eut BMCs to the isolation of recombinant EutSMNLK and EutS compartments from *E. coli* C2566 co-expressing EutC^1–19^-EGFP. As with the *S. enterica* Eut BMCs, a faint UV fluorescent band was detected after sucrose gradient ultracentrifugation and collected for further analysis. SDS-PAGE analysis of the isolated recombinant EutSMNLK shells (**Fig. 5A** **, lane 2**) showed a very similar protein pattern in the expected Eut shell protein size range when compared to the native Eut BMCs (**Fig. 5A** **, lane 1**). Protein patterns differ between the native BMC and recombinant protein shell preparations in the higher molecular weight range. The isolated EutS shell showed one smeared band at around 10 kDa as expected for EutS, but no band corresponding to EutC^1–19^-EGFP. This might be due to a low protein concentration. We noticed that a large fraction of the loaded EutS protein does not migrate into the gel, suggesting that it may retain EutC^1–19^-EGFP, thereby rendering its concentration in the gel too low for detection. Alternatively, lower levels of EutC^1–19^-EGFP could be associated with engineered EutS protein shells which, however, is not supported by results from fluorescent microscopy (**Fig. 4**) and by the detection of a clear fluorescent band comparable to EutSMNLK BMCs during sucrose gradient centrifugation.

TEM analysis of the isolated recombinant EutSMNLK shells showed that they also appeared to be irregular structures (**Fig. 5C**). Purified recombinant EutSMNLK capsids were slightly smaller than the native Eut BMCs (the former are about 100 nm in diameter) (**Fig. 5B and 5C**). This difference in size may represent a difference in the number of different proteins constituting the native and recombinant compartments. While the native Eut shells are believed to be composed of only five shell proteins, a greater number of proteins may be involved in the formation of Eut BMCs in the native *S. enterica* host cell. For example, Pdu BMC shells are composed of seven proteins, one of which was only recently verified as a component of the shell (PduN) [12], [34]. The morphology of purified EutS protein shells was similar to that of EutSMNLK compartments, (**Fig. 5C**), which supports the conclusion that EutS is capable of forming protein shells on its own.

The purification was reproducible and was also applied to the partial purification of native and recombinant Eut shells from cells that do not express EutC^1–19^-EGFP (**Fig. S8B**). Negative stain TEM indicated that the size and morphology of the purified native Eut and recombinant EutSMNLK compartments were unaffected by the presence or absence of the cargo protein EutC^1–19^-EGFP. However, empty EutS compartments (isolated from *E. coli* cells recombinantly expressing EutS only, and not the cargo protein EutC^1–19^-EGFP) appeared to be less able to withstand purification. Isolated empty EutS shells were observed to be 50 nm in diameter, the samples were less homogeneous, and a number of shells appeared to be broken (**Fig. S8B**). Empty EutS shells also sedimented differently from the other protein compartments during sucrose gradient centrifugation.

### Eut BMC-targeted EGFP is sequestered within the Eut microcompartments

**Figure 6:**
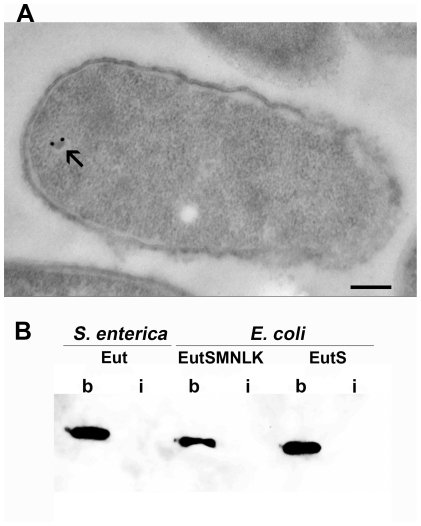
EutC^1–19^-EGFP is sequestered in the recombinant EutSMNLK compartment. (**A**) Anti-GFP immunogold TEM of a thin section of *E. coli* JM109 cells co-expressing EutSMNLK and EutC^1–19^-EGFP. Gold particles are localized to a protein shell. (Scale bar: 200 nm). (**B**) Native gel electrophoresis followed by anti-GFP western blot analysis of broken (b) and intact (i) Eut shells, harboring EutC^1–19^-EGFP.

Anti-GFP immunogold TEM was performed to confirm the localization of EutC^1–19^-EGFP to engineered EutSMNLK shells in permeabilized *E. coli* cells (**Fig. 6A**). As a control, immunofluorescence studies were performed to confirm that the anti-GFP antibody was able to access EutC^1–19^-EGFP in permeabilized *E. coli* cells (**Fig. S9**). As shown in **Fig. 6A**, anti-GFP immunogold beads co-localize at a discrete polyhedral structure located near the pole of the recombinant *E. coli* cell, indicating that EutC^1–19^-EGFP is specifically targeted to the EutSMNLK recombinant compartment. It should be noted that a new protocol (microwave-assisted low temperature processing followed by sectioning and antibody labeling of the permeabilized cells) was developed for our immunogold TEM experiment. Sample preparation for immunogold TEM favored optimal antigenicity but was less than optimal for preserving intracellular structures. The difference in sample preparation protocols can explain the variance in morphology of the shells observed via either immunogold or regular TEM (**Fig. 2**).

We also sought to determine whether EutC^1–19^-EGFP is encapsulated within the Eut shells or if it interacts with the outer surface of the compartments. Eut BMCs isolated from *S. enterica* and Eut shells from *E. coli* (all co-expressing EutC^1–19^-EGFP) were broken by sonication, and intact and broken shells were incubated with anti-GFP antibody using a modified version of the procedure recently described for protein localization into native Pdu BMCs [19]. Western blotting of samples run on a polyacrylamide native gel shows that GFP could be detected in the broken Eut shell preparation (*Salmonella* Eut BMCs as well as recombinant *E. coli* Eut compartments), but not in intact Eut shell preparation (no bands were visible in the high-molecular weight region of the gel) (**Fig. 6B** **and Fig. S10**). These findings indicate that EutC^1–19^EGFP molecules are encapsulated within both native and recombinant Eut compartments (preventing their interactions with anti-GFP antibodies), and are not localized on the outside surface of the Eut protein shells.

### Targeting of an enzyme to the recombinant Eut engineered protein shell

**Figure 7:**
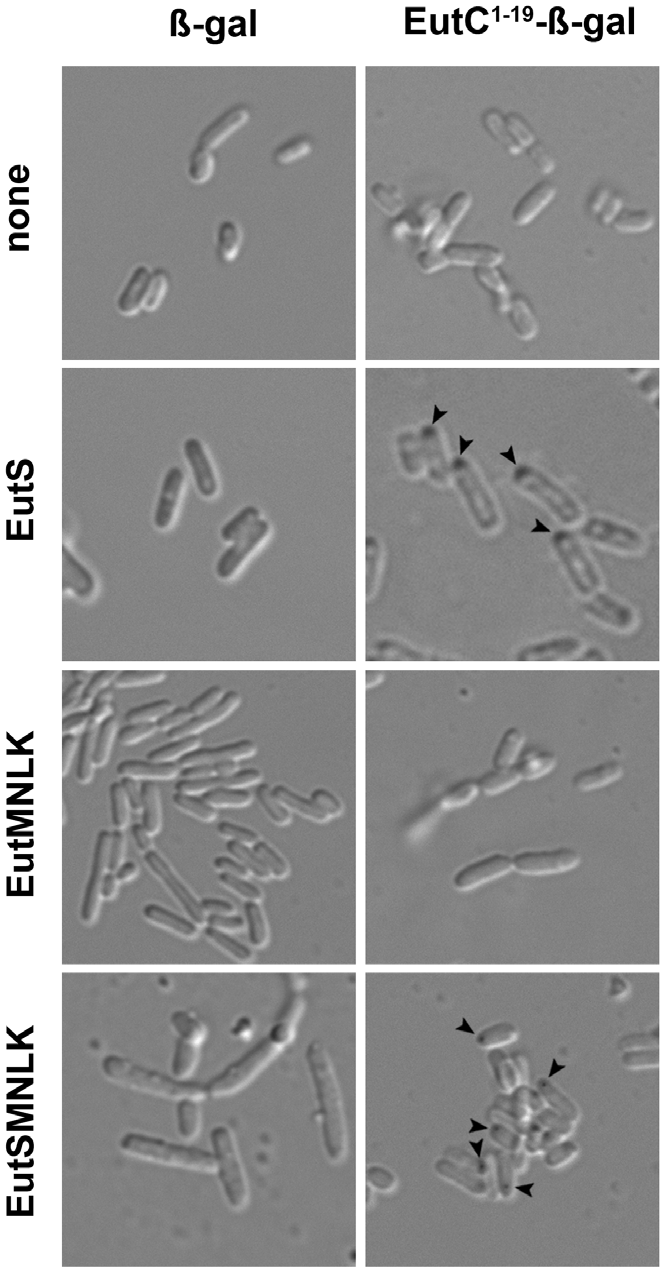
Hydrolysis of X-gal by *E. coli* co-expressing EutC^1–19^-β-galactosidase and recombinant Eut shell proteins. *E. coli* C2566 cells with constructs for constitutive expression of β-galactosidase (β-gal) or EutC^1–19^-β-gal and different combinations of Eut shell proteins were grown with the β-gal substrate X-gal. Intracellular accumulation of the insoluble X-gal cleavage product was observed by Differential Interference Contrast (DIC) microscopy. Arrows point to intracellular indole deposits.

Finally, to explore the feasibility of developing recombinant Eut shells for enzyme sequestration and catalysis, we chose to study hydrolysis of the chromogenic substrate 5-bromo-4-chloro-3-indolyl-β-D-galactopyranoside (X-gal) by β-galactosidase fused N-terminally with the EutC BMC-targeting sequence. Hydrolysis of X-gal produces an insoluble colored indole (indigo), which we hypothesized would form discrete deposits within cells, confirming that EutC^1–19^-ß-galactosidase was targeted to recombinant Eut compartments, and that X-gal could pass into the interior of the capsid. Overnight cultures of *E. coli* cells expressing ß-galactosidase or EutC^1–19^-ß-galactosidase either alone or with different complements of Eut shell proteins (and grown in the presence of X-gal) were all blue, indicating that they possessed functional ß-galactosidase enzyme (data not shown). As shown in **Fig. 7**, *E. coli* co-expressing EutC^1–19^-ß-galactosidase and either EutS or EutSMNLK displayed discrete accumulation of the colored X-gal cleavage product within the cell. The intracellular location of the colored precipitate was compatible with the localization of EutC^1–19^-EGFP, as well as the location of capsid-like structures observed in TEM. No accumulation of the insoluble indigo product was observed within cells co-expressing EutC^1–19^-ß-galactosidase with EutMNLK. Intracellular indole deposits were also absent in cells co-expressing untagged β-galactosidase and Eut shell proteins. Taken together, these data indicate that EutC^1–19^-β-galactosidase is functionally sequestered within the engineered Eut protein shell. The indole deposits in *E. coli* cells expressing EutS appeared to be more diffuse; suggesting that the EutS compartments may be more accessible either due to the presence of only EutS-derived pores or gaps in the protein shell.

## Discussion

Using the Eut BMC shell proteins from *S. enterica* as our model system, we demonstrate that proteinaceous compartments can be engineered in *E. coli*. We also show that heterologous proteins are efficiently targeted into the recombinant compartments; therefore enabling engineering of multi-step biocatalysis within tailored microcompartments as *in vivo* or *in vitro* nano-bioreactors.

In this study, we made the discovery that one of the Eut shell proteins, EutS, is necessary and sufficient for formation of engineered protein shells within *E. coli*. Considering that a 17 gene operon is implicated in the formation of native Eut BMCs, it is surprising that recombinant expression of a single Eut protein (EutS) results in the formation of well-defined compartments that resemble the native *S. enterica* Eut BMCs. Previously, the “bent” hexamer formed by wild type EutS in 3-D crystals had led to the hypothesis that it forms the edges of the BMC shell [28]. Our data indicates that EutS can also form the facets of the capsid, raising the possibility that it adopts more than one conformation *in vivo*. Alternatively, if EutS exists in only one conformation, it can be speculated that a strong reliance on EutS for shell formation may explain why the Eut BMCs are ‘rounder’ than Pdu BMCs or carboxysomes. Remarkably, EutS is also necessary and sufficient for targeting EutC-signal sequence tagged EGFP and β-galactosidase to the recombinant compartment. The EutS-G39V mutant (which forms flat symmetric hexamers) is unable to sequester targeted EGFP, suggesting that the unusual bent configuration adopted by EutS in 3-D crystal lattices is physiologically relevant to the role played by EutS in the engineered compartment [28].

Another significant observation was the successful hydrolysis of X-gal by β-galactosidase localized to the EutS and EutSMNLK compartments, which indicates that these shells allow the indole galactopyranoside to pass through to the interior. The scenarios in which X-gal may gain access to the interior of shells include entry through central pores in shell protein multimers, or via possible gaps between adjacent multimers that form the shell. A large but occluded central pore has been reported in the 3-D crystal structures of EutS and PduU (a close homolog of EutS) [28], [38]. While a substantial rearrangement would be necessary to open the central pore of EutS, it is not without precedent – a recent report demonstrates that exposure to zinc ions causes the central pore of EutL to adopt an “open” conformation [39]. Further studies are required to determine if the central pore in EutS hexamers can also exist in “open” and “closed” conformations. Additionally, two-dimensional (2-D) crystal studies of EutM revealed a tiling pattern with apparent gaps between adjacent hexamers, a phenomenon not observed in the 3-D crystal structure [28], [39]. This discrepancy raises the question if EutS hexamers, and indeed other Eut BMC proteins, can also form 2-D lattices which are not as tightly packed as those observed in their 3-D crystals.

The ability of EutS to self-assemble into compartments and sequester targeted proteins within its interior raises questions about the physiological roles of the other Eut shell proteins. It is possible that the EutM, EutN, EutL and EutK shell proteins impart selectivity in terms of the small molecules allowed to enter or exit the BMC shell. For example, EutL crystallizes as a pseudohexamer with a large, gated central pore that may play an important role in transporting substrates and bulky cofactors into the shells while preventing the loss of reaction intermediates [28], [39]. Incorporation of EutMNLK may also increase the stability of the microcompartments. Other possible roles of EutMNLK in the native *S. enterica* Eut BMCs include interactions with the encapsulated ethanolamine utilization enzymes, thereby regulating their spatial organization within the compartment [18]. Further biochemical studies are required to elucidate the roles played by Eut shell proteins in BMC organization and function. The heterologous reconstitution of Eut compartments provides an important tool for such studies.

Bobik and colleagues had predicted the presence of N-terminal signal sequences on EutC and EutG enzymes [19]. Our experiments indicate that the first nineteen amino acids of EutC (but not EutG) are sufficient to target heterologous proteins to native Eut BMCs in *Salmonella* as well as recombinant Eut compartments in *E. coli*. The reasons behind the inability of EutG^1–19^ to function as a BMC-targeting sequence are unclear. It is possible that steric hindrance prevents EutG^1–19^ from functioning as a BMC-targeting sequence, and extending the length of the EutG sequence by a few amino acids may reduce the hindrance and allow it to function as a targeting signal. An additional example is the PduC enzyme, which was predicted to lack a BMC-targeting sequence [19]. However, a separate study showed that recombinant PduC-GFP fusion protein displays punctate localization when co-expressed with Pdu shell proteins, suggesting that it is targeted to recombinant Pdu compartments [30]. These observations suggest that there may be multiple mechanisms for targeting proteins for encapsulation within BMCs, and further experiments are required to understand them.

The functions of several genes in the *eut* operon are at present unknown. While *S. enterica* cells contained multiple Eut BMCs distributed throughout the cytoplasm, recombinant compartments in *E. coli* were restricted to only one or two per cell. Some of the non- BMC-shell *eut* genes may be required for the formation of more than one microcompartment per cell, through for instance, transcriptional regulation [40]. The off-center location of the recombinant compartments was also invariant. A recent report indicates that cyanobacterial carboxysomes are in motion within the cell and interact with the cytoskeleton, thereby ensuring their equitable distribution during cell division [41]. If one or more of the non-BMC-shell Eut proteins (for example EutP, an Era (*E. coli* Ras-like protein)-like GTPase) are required for interacting with the bacterial cytoskeleton, the intracellular location of the recombinant shell may simply result from aberrant interactions with the cytoskeleton leading to altered nucleation of the microcompartment. Our observation that native Eut BMCs exhibit movement in *S. enterica*, while recombinant Eut compartments appear to be stationary in *E. coli*, lends credence to this hypothesis. Further investigations into the role of as yet uncharacterized proteins encoded by BMC operons should provide important insights into BMC nucleation, assembly and cytoskeletal interaction, and may present a handle for controlling the number of recombinant compartments formed inside engineered cells.

While this manuscript was under review, heterologous expression of *Halothiobacillus neapolitanus* carboxysomes in *E. coli* was demonstrated by Bonacci *et al* [42]. Expression of several shell proteins appears to be required for the formation of carboxysomes, whereas EutS alone is sufficient to form heterologous protein shells. Compared to carboxysomes, a EutS-based system is less complex, and may find broader applicability in the engineering of metabolic pathways and multi-enzymatic biocatalysis. Our results show that Eut protein shells can be engineered to sequester heterologous enzymes for catalysis. A recent report outlines a different approach for enzyme encapsulation - addition of oppositely charged amino acids to a non-BMC shell protein (lumazine synthase) and the encapsulated enzyme [43]. Compared to this latter strategy, which involves optimization of electrostatic interactions between shell and cargo proteins while maintaining the catalytic efficiency of each sequestered enzyme, the *in vivo* system offered by EutS and the nineteen amino acid BMC targeting sequence is much simpler, and very specific.

Next steps in the engineering of microcompartments will include encapsulation of multiple enzymes for biosynthesis or biodegradation with engineered cells, and in addition, *in vitro* multi-step catalysis with isolated and immobilized compartments. The central pores formed by multimers of BMC shell proteins offer opportunities to engineer such protein nano-bioreactors with desired selectivities for substrates and products. Additionally, recombinant hybrid compartments may be formed by combining shell proteins from different BMC types and sources. In the future, synthetic biologists may be able to design protein nano-bioreactors to their desired specifications by selecting from a rapidly expanding library of native and engineered BMC shell proteins as well as targeting sequences. The results presented in this study bring us an important step closer to the rational design of protein compartments useful for a variety of *in vivo* and *in vitro* applications.

## Methods

### Microbiological methods

*E. coli* cultures were cultivated aerobically at 30°C for 15–18 h in Luria-Bertani (LB) medium supplemented with the appropriate antibiotic when required (ampicillin 100 µg ml^−1^, chloramphenicol 50 µg ml^−1^). For the *in vivo* β-galactosidase assay, cultures were grown overnight with X-gal (final concentration: 0.008% (w/v)). *S. enterica* cultures were grown aerobically at 37°C overnight in supplemented E medium with 150 nM vitamin B_12_ (cyanocobalamin) and either 0.2% (v/v) glycerol or 30 mM ethanolamine [17]. 30 µg ml^−1^ kanamycin was added to the growth media for culturing *S. enterica* strains containing pBBRBB-based plasmids.

### Gene cloning

Eut shell genes were amplified from *S. enterica* genomic DNA with gene specific primers containing suitable restriction sites. Their accession numbers are as follows: EutS (NP_461405), EutM (NP_461400), EutN (NP_461399), EutL (NP_461391), and EutK (NP_461390). The *eut* genes were cloned into our in-house BioBrick™ expression vector pUCBB, and expressed from a constitutively active modified *lac* promoter described previously (**Fig. S1**) [44], [45]. The EutS-G39V mutant was created by site-directed mutagenesis. Additional details of the cloning strategy are presented in **Methods S1**. β-galactosidase (amplified from *E. coli* MG1655 genomic DNA) and EGFP were cloned into our in-house low copy number BioBrick™ expression vector pACBB. Nucleotides coding for the putative EutC-signal sequence (EutC^1–19^) from *S. enterica* LT2 (MDQKQIEEIVRSVMASMGQ) were added to N-terminus of EGFP and β-galactosidase through PCR [19]. Similarly, EGFP was also tagged with the predicted EutG-signal sequence (EutG^1–19^) from *E. coli* K12 (MQNELQTALFQAFDTLNLQ), which differs from the *S. enterica* LT2 EutG^1–19^ by one amino acid. Both putative signal sequences were codon optimized for expression in *E. coli*.

### Transmission Electron Microscopy (TEM)

Bacterial cells were fixed in 2.5% glutaraldehyde in 0.1 M phosphate buffer, followed by three washes with 0.1 M phosphate buffer. Triton X-100 was added to the glutaraldehyde solution and rinse buffer to a final concentration of 0.1%. Subsequently, the pellets were post-fixed with 1% osmium tetroxide in 0.1 M phosphate buffer, washed with nanopure water, and embedded in 2% low melting agarose. The cell-agarose pellet was cut into 1 mm^3^ cubes, and dehydrated using an ethanol gradient. The cell-agarose cubes were then incubated in 1∶1 mixture of Embed 812 resin and 100% ethanol for 4 h, followed by 18 h incubation in 100% Embed 812 resin. Next, they were suspended in a fresh Embed 812 resin-N,N-dimethylbenzylamine (BDMA) solution and polymerized at 60°C for 48 h. 90 nm sections were sliced, placed on 200 mesh formvar-coated copper grids, and post-stained with 3% uranyl acetate and Triple lead stain. Specimens were observed and photographed with a Philips CM12 transmission electron microscope.

### Light Microscopy

Bacteria were viewed using a Nikon Eclipse E800 photomicroscope equipped with bright field, Differential Interference Contrast (DIC) and fluorescence optics including blue (excitation filter 470–490 nm, barrier 520–580 nm) and green (excitation filter 510–560 nm, barrier 570–620 nm) filter sets. The samples were viewed using a 100×, 1.4 n.a. plan apo objective. For fluorescence microscopy, 16-bit digital images were collected using a Roper CoolSnap HQ monochrome camera and captured using Image Pro Plus software. DIC microscopy was performed using a 1.4 n.a. oil condenser. Z-series images of cells were collected at 0.15 micron steps using a Ludl MAC 3000 controller interfaced with ImagePro Plus. The DIC images were deconvolved using the SharpStack Nearest Neighbor algorithm. A minimum projection of the resulting z-series was made and all images were identically adjusted for display using PhotoShop.

### Purification of protein compartments

*S. enterica* cells were made electrocompetent, and were transformed with pACBBEutC^1–19^-EGFP. Cells harboring the plasmid were grown in 1 liter of NCE minimal medium supplemented with 50 µg ml^−1^ chloramphenicol and 30 mM ethanolamine to induce Eut BMC production [46]. *E. coli* C2566 cells harboring pUCBBEutSMNLK or pUCBBEutS were also transformed with pACBBEutC^1–19^-EGFP and were grown in 1 liter of LB medium supplemented with 100 µg ml^−1^ ampicillin and 50 µg ml^−1^ chloramphenicol. *E. coli* C2566 cells harboring pACBBEutC^1–19^-EGFP were also grown as a control. Cultures were grown at 37°C with shaking at 275 rpm for 16 h. Cells were harvested by centrifugation at 10, 000×g for 30 min at 4°C. Purification of Eut shells was carried out using procedures previously described for Pdu BMCs, with the exception that the TEMP buffer was replaced by a TEME buffer (50 mM Tris.HCl, 10 mM MgCl_2_, 2 mM EDTA, 30 mM ethanolamine (pH 8.0)), and that the shells were applied to two separate discontinuous sucrose gradients: firstly, a three step gradient of 20, 40 and 65% sucrose, and secondly a ten step gradient from 22 to 54% sucrose [13]. Native Eut BMCs from *S. enterica* harboring EutC^1–19^-EGFP and recombinant EutSMNLK and EutS shells from *E. coli* harboring EutC^1–19^-EGFP formed a white translucent band two thirds the way down the centrifuge tube, which was also checked for fluorescence under UV light due to the localization of EutC^1–19^-EGFP into the BMCs. Following a final clarification step, the native Eut BMCs and recombinant EutSMNLK and EutS shells were pelleted, and sample purity was judged to be sufficient for TEM by SDS-PAGE electrophoresis. Purified shell samples were fixed and negatively stained according to previously published procedures, with the exception that 2% uranyl acetate was used for staining [13]. The purification and negative staining procedures were repeated on separate occasions to ensure consistency in results and reproducibility of the procedure.

### Anti-GFP Immunogold labeling and TEM

Microwave-assisted low temperature processing was used to prepare *E. coli* cells for anti-GFP immunofluorescence and immunogold labeling. Steps for primary fixation of the samples were adapted from procedures published previously [47], [48]. The samples underwent dehydration/substitution in methanol/LR white. Thin sections were placed on formvar-coated nickel 200 mesh grids followed by anti-GFP immunogold labeling. Additional experimental details are included in **Methods S1**.

**Additional methods** including SDS-PAGE electrophoresis and western blotting are described in **Methods S1**.

## Supporting Information

Figure S1 **BioBrick™ vectors and strategy for stacking multiple genes into a single plasmid.** (**A**) Our in-house BioBrick™ vectors contain an expression cassette with a constitutive promoter (P_lac*_) and an EGFP reporter. (**B**) Cloning of Eut BMC shell genes into pUCBB. (i) EutS, EutMN and EutLK were cloned downstream of the constitutive P_lac*_ promoter (blue arrow) using BglII and NotI. (ii) and (iii) Expression cassettes for EutMNLK and EutSMNLK were created as described in **Methods S1**.
(TIF)

Figure S2 **SDS/PAGE analysis showing recombinant expression of** ***S. enterica*** **Eut shell proteins in** ***E. coli*** **.** (**A**) Overexpression of Eut shell proteins in the *E. coli* strain C2566. (**B**) Overexpression of Eut shell proteins in the *E. coli* strain JM109. (**c**) Overexpression of wild type EutS and the EutS-G39V mutant in *E. coli* strains C2566 and JM109. 15 µg soluble protein fraction was loaded in each lane. Expected protein sizes are as follows: EutS (11.6 kDa), EutM (9.8 kDa), EutN (10.4 kDa), EutL (22.7 kDa), EutK (17.5 kDa), and EGFP (26.9 kDa). Proteins were stained with Coomassie Blue.
(TIF)

Figure S3 **Transmission electron micrographs of thin sections of recombinant** ***E. coli*** **expressing** ***S. enterica*** **Eut shell proteins.** (**A–C**) *E. coli* expressing recombinant EutS contain properly delimited shells (*E. coli* strain used in **A**: C2566, and in **B**, **C**: JM109). (**D–F**) *E. coli* expressing recombinant EutM form thick axial filaments that interfere with separation after cell-division (*E. coli* strain used in **D, E**: C2566, and in **F**: JM109). (**G**) *E. coli* JM109 expressing recombinant EutN. (**H**) *E. coli* JM109 expressing recombinant EutL. (**I**) *E. coli* JM109 expressing recombinant EutK shows an electron translucent region in the middle of the cell. (**J**) An electron dense region is visible in *E. coli* JM109 co-expressing recombinant EutM and EutN. (**K**) Intracellular filaments are formed in *E. coli* JM109 co-expressing recombinant EutL and EutK. (**L–N**) Clearly defined shells are observed in *E. coli* JM109 expressing recombinant EutSMNLK. (**O–Q**) Co-expression of EutSMNLK and EutC^1–19^-EGFP results in the formation of compartments that are morphologically similar to the shells observed *in vivo* by expression of either EutS or EutSMNLK alone. (*E. coli* strain used in **O**: C2566, and in **P, Q**: JM109). Arrows indicate the location of recombinant shells. (Scale bar: 200 nm).
(TIF)

Figure S4 **Localization of EutC^1–19^-EGFP in recombinant** ***E. coli*** **JM109 cells expressing** ***S. enterica*** **Eut shell proteins.** Fluorescence microscopy images of *E. coli* JM109 cells co-expressing EGFP or EutC^1–19^-EGFP with EutS (wild type and the G39V mutant), EutMNLK or EutSMNLK. See **Table S2** for the quantification of EGFP localization in recombinant *E. coli*, and **Fig. 4** for the localization of EutC^1–19^-EGFP in the *E. coli* C2566 strain. Cell boundaries are shown by the DIC images.
(TIF)

Figure S5 **Localization of EutC^1–19^-EGFP in recombinant** ***E. coli*** **C2566 cells expressing various combinations of** ***S. enterica*** **Eut shell proteins.** Fluorescence microscopy images of *E. coli* C2566 cells with constructs for constitutive expression of EGFP or EutC^1–19^-EGFP with EutM, EutN, EutL, EutK, EutMN and EutLK. In the absence of EutS, there is no discrete fluorescent localization of EutC^1–19^-EGFP, which indicates that EutS is required for targeting EutC^1–19^-EGFP to the engineered microcompartments. Cell boundaries are shown by the DIC images.
(TIF)

Figure S6 **Nile Red staining of recombinant** ***E. coli*** **expressing EutC^1–19^-EGFP.** *E. coli* C2566 cells co-expressing EutC^1–19^-EGFP and EutS or EutSMNLK were stained with the fluorescent, lipophilic inclusion body stain Nile Red. Co-localization of red and green fluorescence was not observed, indicating that the recombinant Eut shells are not inclusion bodies nor are the surrounded by a hydrophobic matrix.
(TIF)

Figure S7 **Nile Red staining of recombinant** ***E. coli*** **expressing NSC1.** *E. coli* C2566 cells co-expressing the cyanobacterial carotenoid cleavage dioxygenase NSC1 either alone or with EutC^1–19^-EGFP. While red fluorescent puncta corresponding to inclusion bodies were observed in the presence of NSC1, co-localization of red and green fluorescence was not seen, showing that EutC^1–19^-EGFP is not targeted to NSC1 inclusion bodies.
(TIF)

Figure S8 **Transmission electron micrographs of partially purified protein compartments.** (**A**) Native Pdu BMCs isolated from *S. enterica*. (**B**) Native Eut BMCs and recombinant Eut protein shells isolated from cells not expressing the cargo protein EutC^1–19^-EGFP. From left to right: Native Eut BMCs isolated from *S. enterica*, recombinant EutSMNLK shells isolated from *E. coli* C2566, and recombinant EutS shells isolated from *E. coli* C2566. Scale bar: 100 nm.
(TIF)

Figure S9 **Immunofluorescence analysis of EutC^1–19^-EGFP localization in recombinant** ***E. coli*** **expressing Eut shell proteins.** EGFP, anti-GFP antibody (red) and merged EGFP-anti-GFP antibody fluorescence signals from *E. coli* cells with constructs for constitutive expression of EGFP or EutC^1–19^-EGFP with EutS or EutSMNLK. (**A**) anti-GFP immunofluorescence studies in the *E. coli* strain C2566. (**B**) anti-GFP immunofluorescence studies in the *E. coli* strain JM109.
(TIF)

Figure S10 **Separation of EutC^1–19^-EGFP from broken and intact Eut shells by native polyacrylamide electrophoresis.** Visualization of protein migration by silver stain of native gel. EGFP control is shown in lane 1, followed by broken (lane 2) and intact (lane 3) Eut BMCs from *S. enterica* cells harboring EutC^1–19^-EGFP; broken (lane 4) and intact (lane 5) recombinant EutSMNLK BMCs co-expressing EutC^1–19^-EGFP; and broken (lane 6) and intact (lane 7) recombinant EutS BMCs from *E. coli* C2566 cells co-expressing EutC^1–19^-EGFP.
(TIF)

Video S1 **Dynamics of EutC^1–19^-EGFP in** ***S. enterica*** **grown on ethanolamine.** Representative time-lapse movie of *S. enterica* cells harboring pBBRBB-EutC^1–19^-EGFP, and grown in the presence of ethanolamine. Discrete fluorescent foci are observed to be in motion within the *S. enterica* cells, suggesting that Eut BMCs (which would be expected to encapsulate EutC^1–19^-EGFP) are moving around within the cell. Time stamp on video indicates elapsed time. Preparations were viewed using a Nikon Eclipse E800 photomicroscope. Time-lapse images were collected at 15 second intervals. Shutters were opened only during camera exposure.
(AVI)

Table S1 **Quantification of the distribution of recombinant Eut shells in** ***E. coli*** **.** Thin cell sections of *E. coli* expressing EutS or EutSMNLK were observed by TEM. Assuming an average *E. coli* cell has a height of 2 µm and a diameter of 0.5 µm, about 20 thin sections (90 nm in width) perpendicular to the axis can be cut from each cell. An average recombinant Eut protein shell has a diameter of 100–200 nm. The average *E. coli* cell would have about 20 cross-sections parallel to the circular base, of which only two would pass through an engineered Eut shell. Even if 100% of *E. coli* had a recombinant Eut compartment, the actual fraction of cell cross-sections showing the phenotype would be around 10%. For sections parallel to the height of the cell, less than 40% would be expected to display the shell, and the number showing compartments at close to their maximum width will be even lower.
(DOC)

Table S2 **Quantification of the distribution of engineered Eut protein shells in** ***E. coli*** **by fluorescence microscopy.**
(DOC)

Methods S1 **Supporting Methods.**
(DOC)
