# Supplementary figures and images for: Engineered Protein Nano-Compartments for Targeted Enzyme Localization
Source: PLoS One. 2012 Mar 12;7(3):e33342. doi: 10.1371/journal.pone.0033342 (PMC3299773; doi:10.1371/journal.pone.0033342)

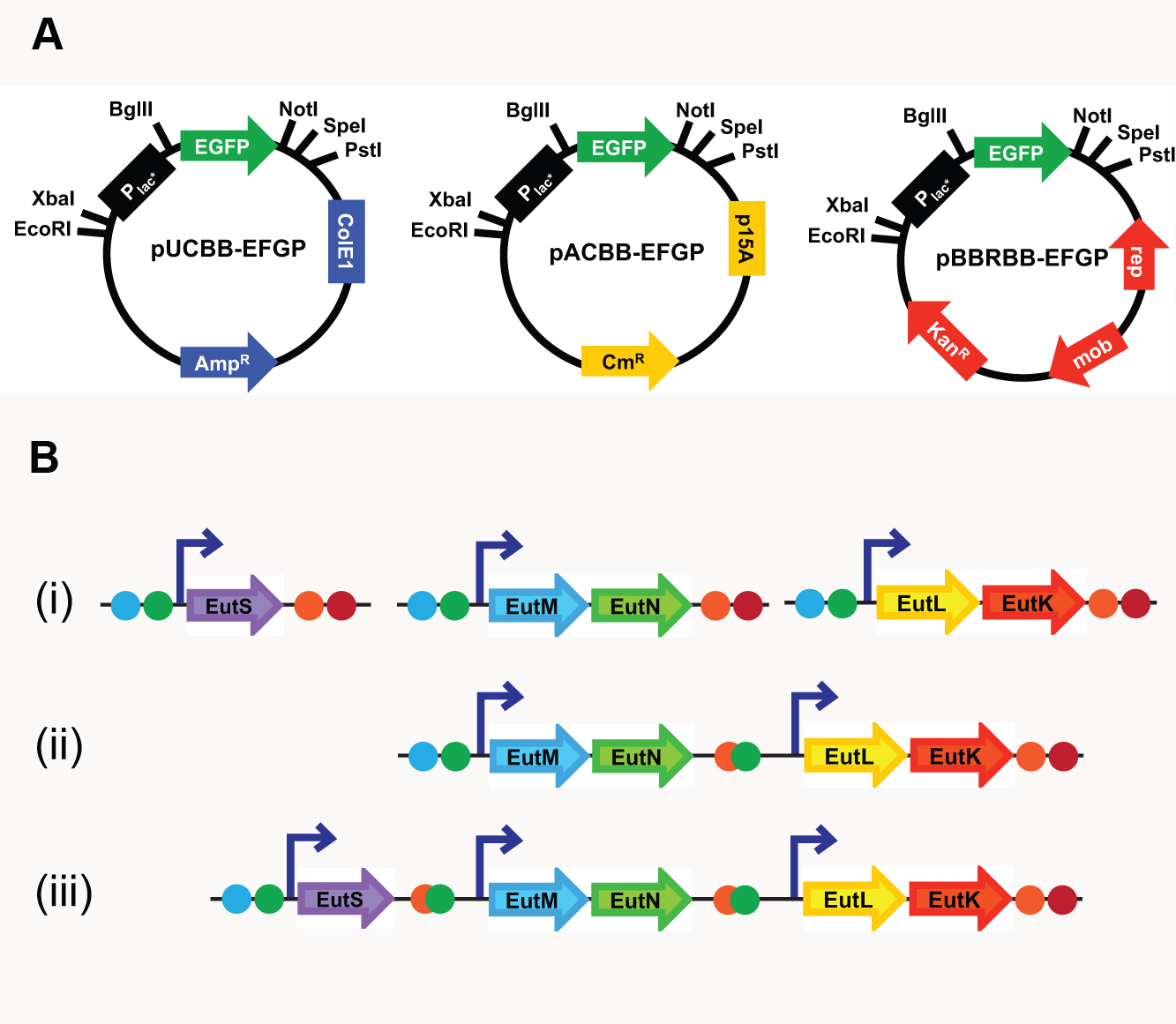

Supplement: Figure S1 — BioBrick™ vectors and strategy for stacking multiple genes into a single plasmid. (A) Our in-house BioBrick™ vectors contain an expression cassette with a constitutive promoter (Plac*) and an EGFP reporter. (B) Cloning of Eut BMC shell genes into pUCBB. (i) EutS, EutMN and EutLK were cloned downstream of the constitutive Plac* promoter (blue arrow) using BglII and NotI. (ii) and (iii) Expression cassettes for EutMNLK and EutSMNLK were created as described in Methods S1. (TIF) [file pone.0033342.s001.tif]

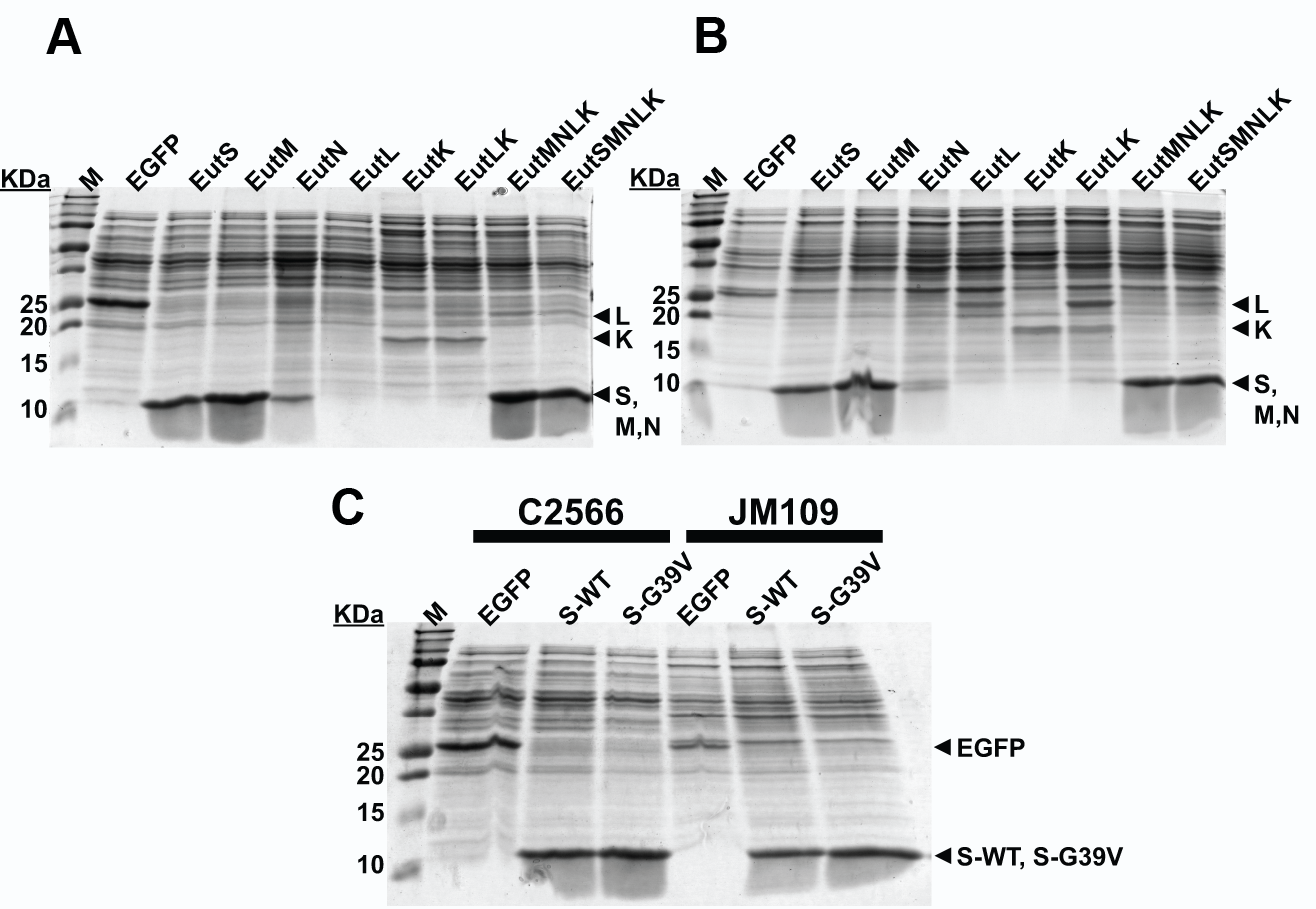

Supplement: Figure S2 — SDS/PAGE analysis showing recombinant expression of S. enterica Eut shell proteins in E. coli . (A) Overexpression of Eut shell proteins in the E. coli strain C2566. (B) Overexpression of Eut shell proteins in the E. coli strain JM109. (c) Overexpression of wild type EutS and the EutS-G39V mutant in E. coli strains C2566 and JM109. 15 µg soluble protein fraction was loaded in each lane. Expected protein sizes are as follows: EutS (11.6 kDa), EutM (9.8 kDa), EutN (10.4 kDa), EutL (22.7 kDa), EutK (17.5 kDa), and EGFP (26.9 kDa). Proteins were stained with Coomassie Blue. (TIF) [file pone.0033342.s002.tif]

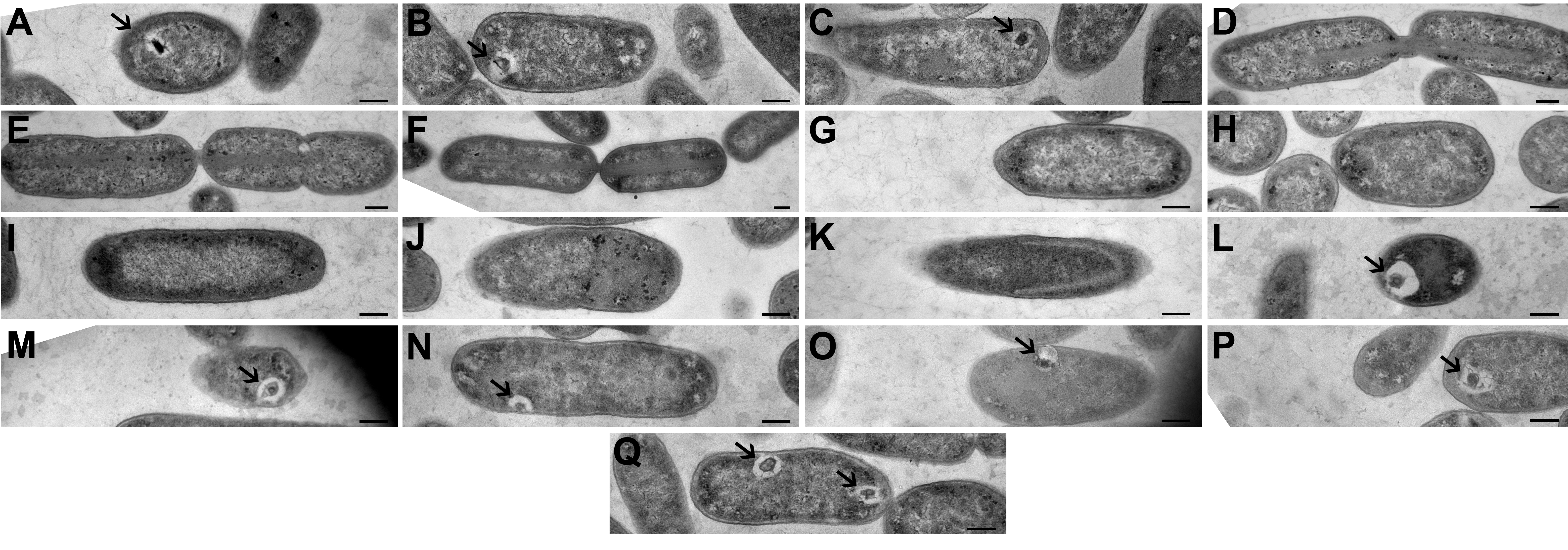

Supplement: Figure S3 — Transmission electron micrographs of thin sections of recombinant E. coli expressing S. enterica Eut shell proteins. (A–C) E. coli expressing recombinant EutS contain properly delimited shells (E. coli strain used in A: C2566, and in B, C: JM109). (D–F) E. coli expressing recombinant EutM form thick axial filaments that interfere with separation after cell-division (E. coli strain used in D, E: C2566, and in F: JM109). (G) E. coli JM109 expressing recombinant EutN. (H) E. coli JM109 expressing recombinant EutL. (I) E. coli JM109 expressing recombinant EutK shows an electron translucent region in the middle of the cell. (J) An electron dense region is visible in E. coli JM109 co-expressing recombinant EutM and EutN. (K) Intracellular filaments are formed in E. coli JM109 co-expressing recombinant EutL and EutK. (L–N) Clearly defined shells are observed in E. coli JM109 expressing recombinant EutSMNLK. (O–Q) Co-expression of EutSMNLK and EutC1–19-EGFP results in the formation of compartments that are morphologically similar to the shells observed in vivo by expression of either EutS or EutSMNLK alone. (E. coli strain used in O: C2566, and in P, Q: JM109). Arrows indicate the location of recombinant shells. (Scale bar: 200 nm). (TIF) [file pone.0033342.s003.tif]

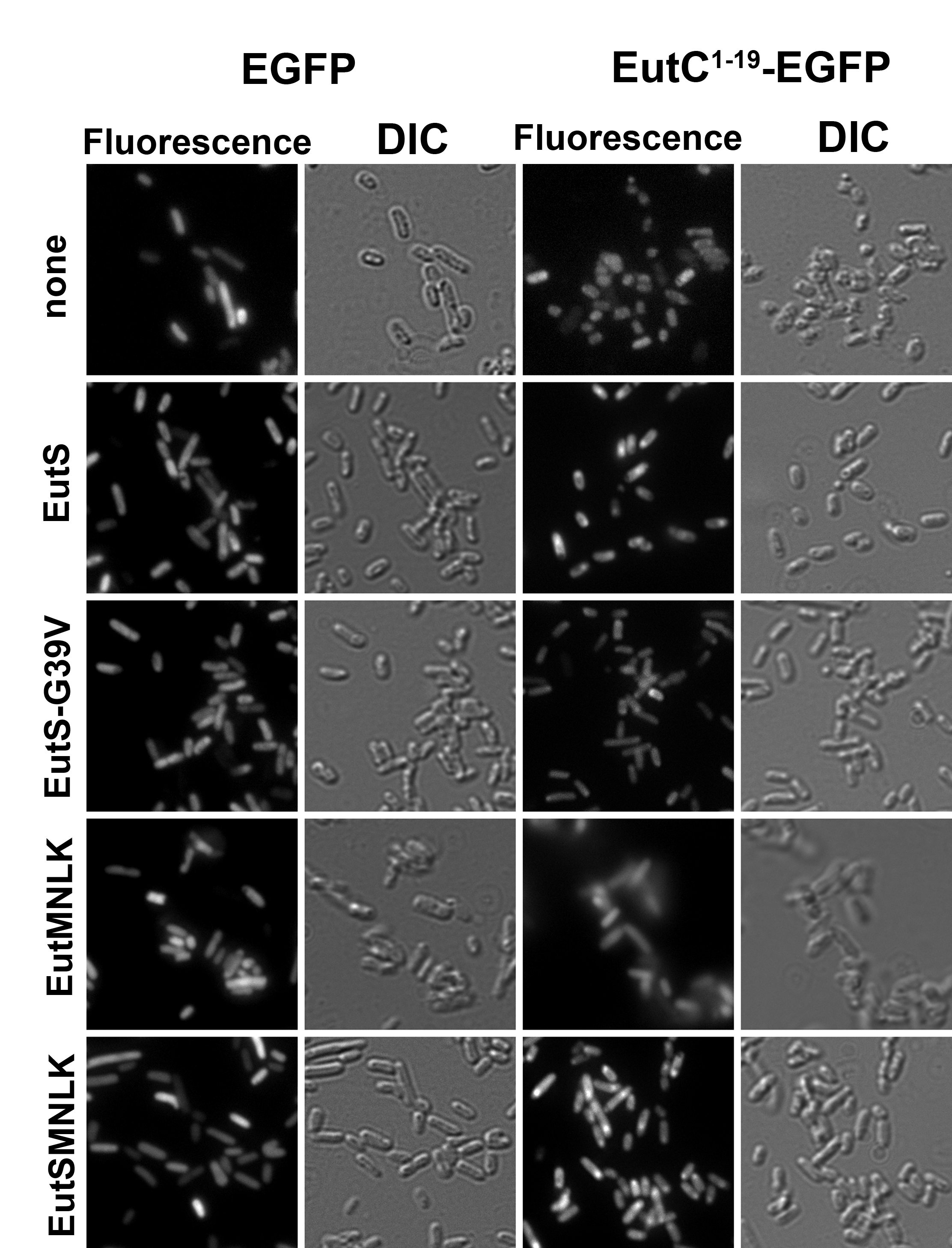

Supplement: Figure S4 — Localization of EutC1–19-EGFP in recombinant E. coli JM109 cells expressing S. enterica Eut shell proteins. Fluorescence microscopy images of E. coli JM109 cells co-expressing EGFP or EutC1–19-EGFP with EutS (wild type and the G39V mutant), EutMNLK or EutSMNLK. See Table S2 for the quantification of EGFP localization in recombinant E. coli, and Fig. 4 for the localization of EutC1–19-EGFP in the E. coli C2566 strain. Cell boundaries are shown by the DIC images. (TIF) [file pone.0033342.s004.tif]

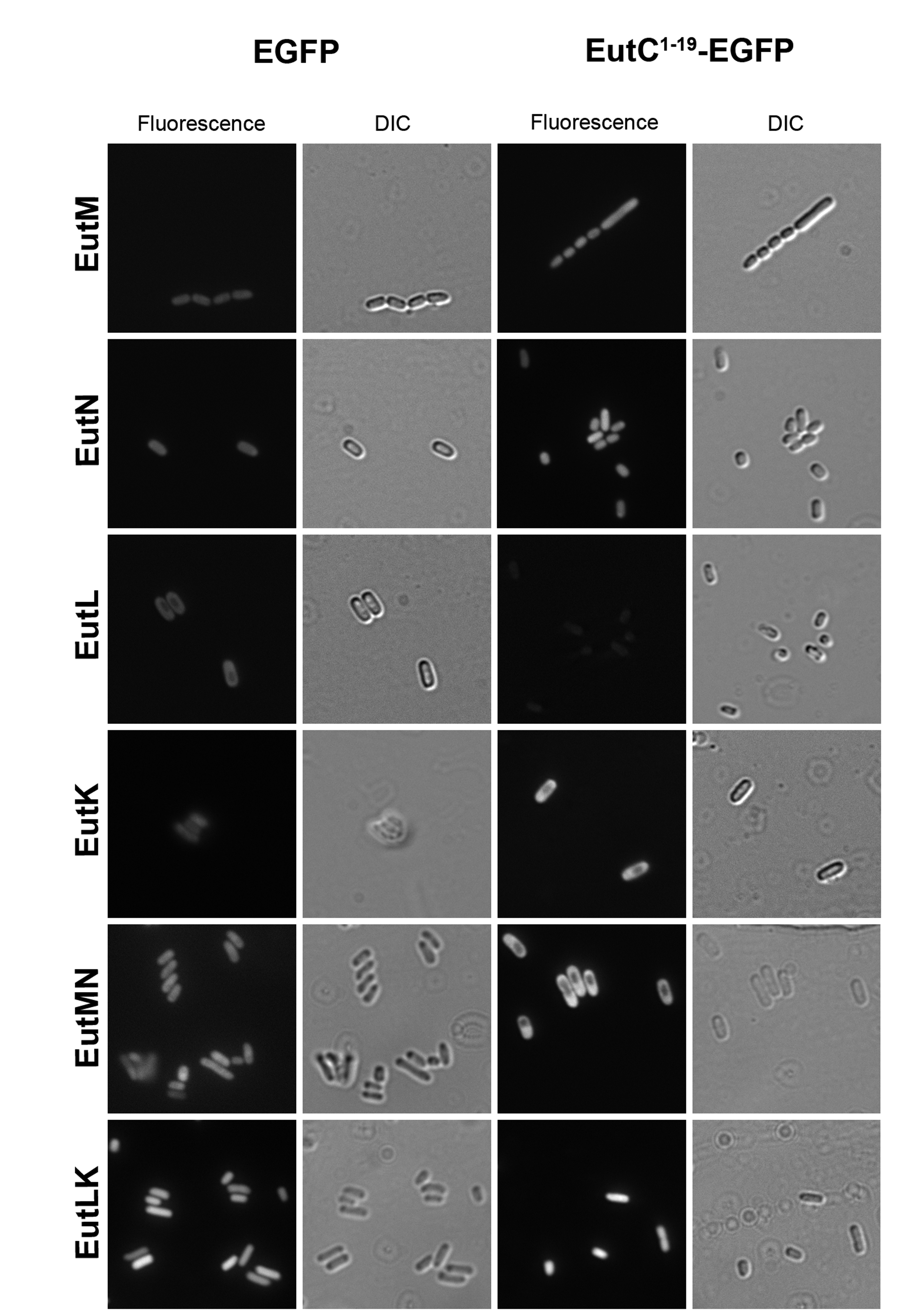

Supplement: Figure S5 — Localization of EutC1–19-EGFP in recombinant E. coli C2566 cells expressing various combinations of S. enterica Eut shell proteins. Fluorescence microscopy images of E. coli C2566 cells with constructs for constitutive expression of EGFP or EutC1–19-EGFP with EutM, EutN, EutL, EutK, EutMN and EutLK. In the absence of EutS, there is no discrete fluorescent localization of EutC1–19-EGFP, which indicates that EutS is required for targeting EutC1–19-EGFP to the engineered microcompartments. Cell boundaries are shown by the DIC images. (TIF) [file pone.0033342.s005.tif]

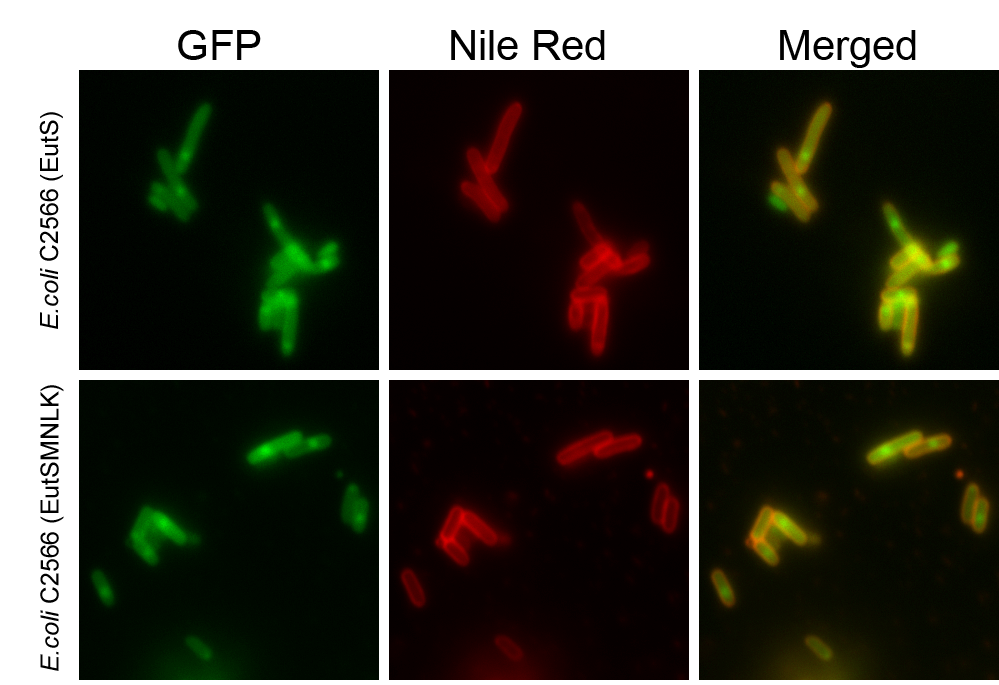

Supplement: Figure S6 — Nile Red staining of recombinant E. coli expressing EutC1–19-EGFP. E. coli C2566 cells co-expressing EutC1–19-EGFP and EutS or EutSMNLK were stained with the fluorescent, lipophilic inclusion body stain Nile Red. Co-localization of red and green fluorescence was not observed, indicating that the recombinant Eut shells are not inclusion bodies nor are the surrounded by a hydrophobic matrix. (TIF) [file pone.0033342.s006.tif]

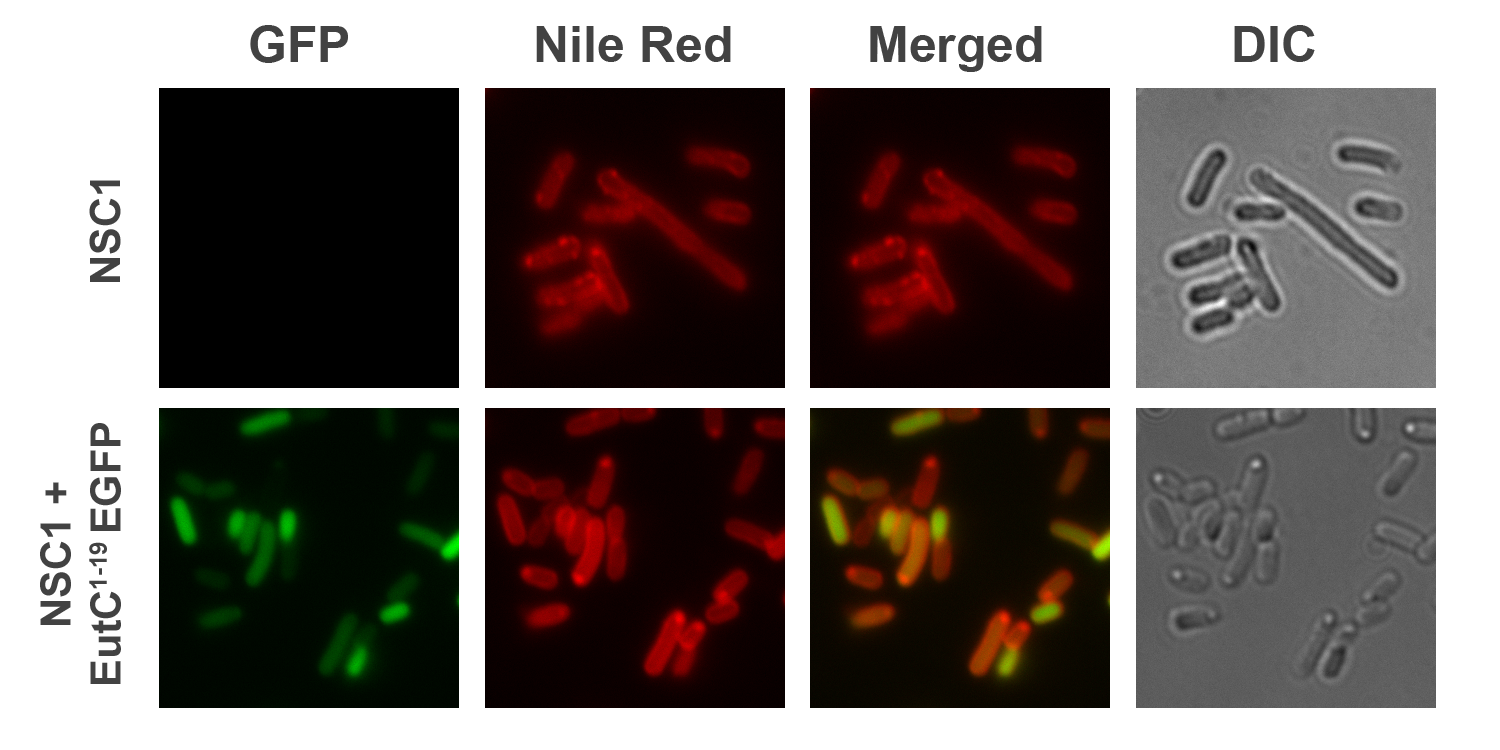

Supplement: Figure S7 — Nile Red staining of recombinant E. coli expressing NSC1. E. coli C2566 cells co-expressing the cyanobacterial carotenoid cleavage dioxygenase NSC1 either alone or with EutC1–19-EGFP. While red fluorescent puncta corresponding to inclusion bodies were observed in the presence of NSC1, co-localization of red and green fluorescence was not seen, showing that EutC1–19-EGFP is not targeted to NSC1 inclusion bodies. (TIF) [file pone.0033342.s007.tif]

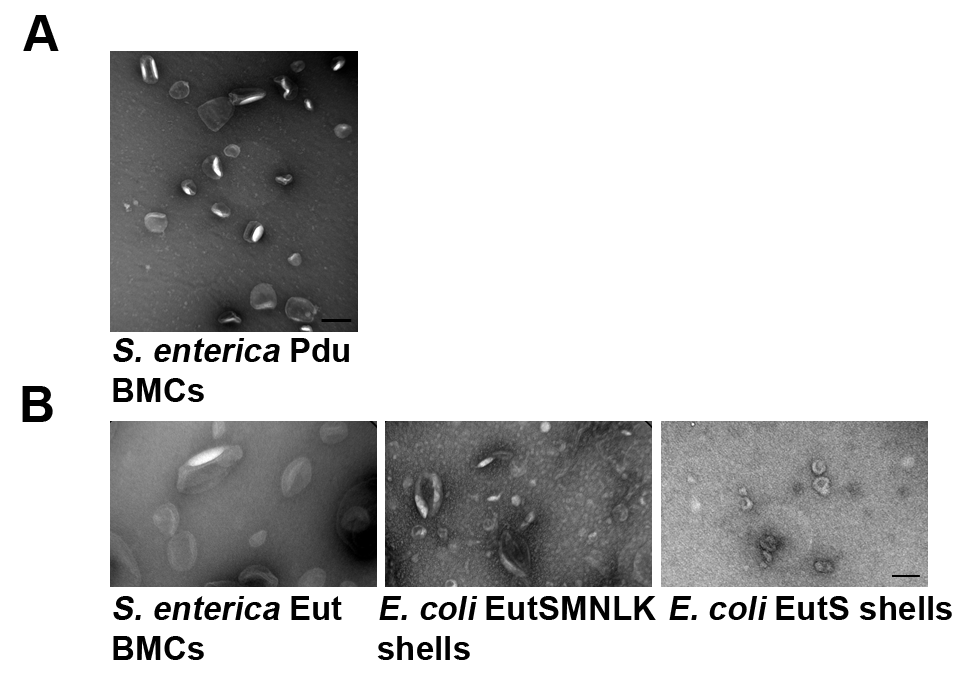

Supplement: Figure S8 — Transmission electron micrographs of partially purified protein compartments. (A) Native Pdu BMCs isolated from S. enterica. (B) Native Eut BMCs and recombinant Eut protein shells isolated from cells not expressing the cargo protein EutC1–19-EGFP. From left to right: Native Eut BMCs isolated from S. enterica, recombinant EutSMNLK shells isolated from E. coli C2566, and recombinant EutS shells isolated from E. coli C2566. Scale bar: 100 nm. (TIF) [file pone.0033342.s008.tif]

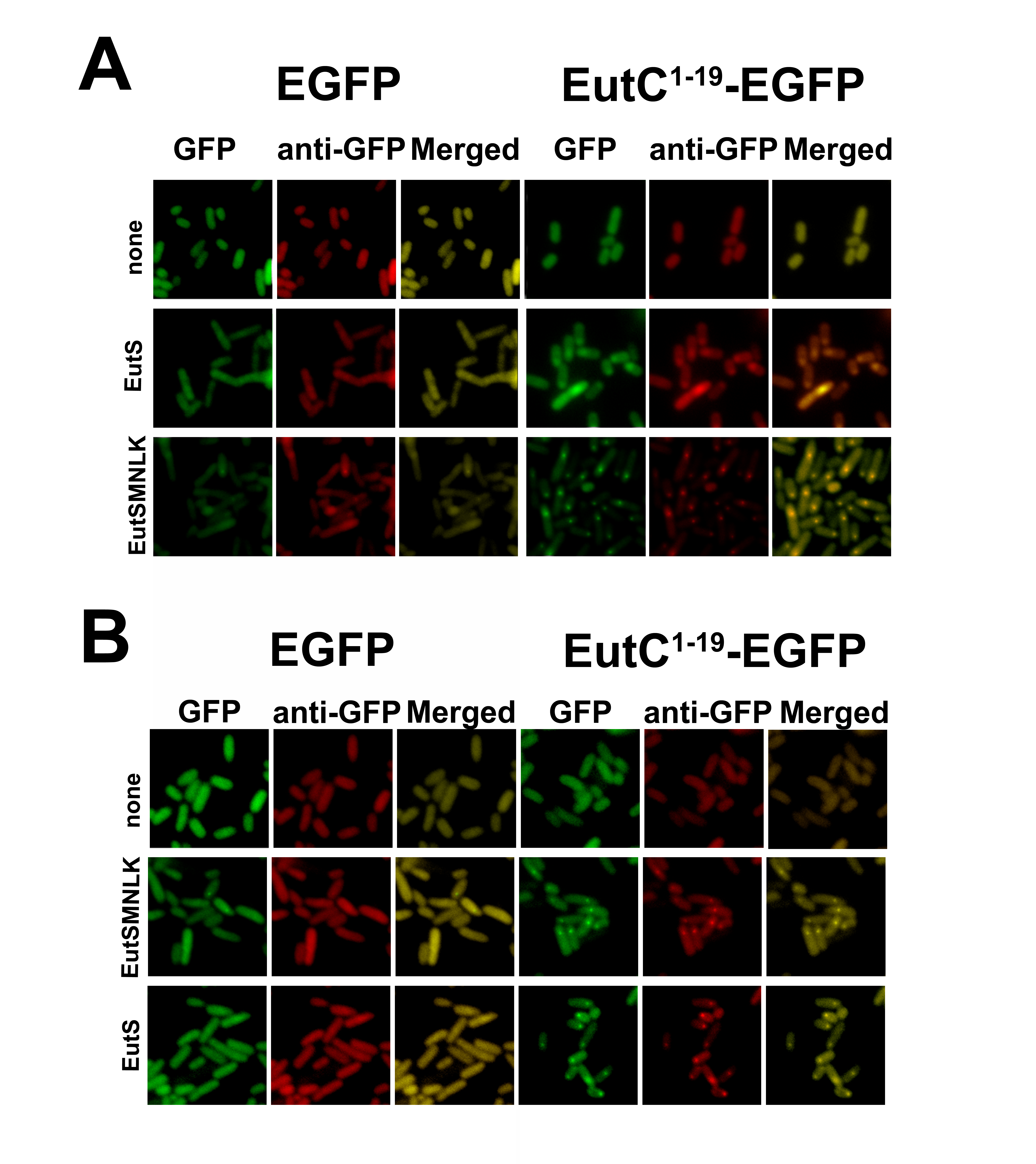

Supplement: Figure S9 — Immunofluorescence analysis of EutC1–19-EGFP localization in recombinant E. coli expressing Eut shell proteins. EGFP, anti-GFP antibody (red) and merged EGFP-anti-GFP antibody fluorescence signals from E. coli cells with constructs for constitutive expression of EGFP or EutC1–19-EGFP with EutS or EutSMNLK. (A) anti-GFP immunofluorescence studies in the E. coli strain C2566. (B) anti-GFP immunofluorescence studies in the E. coli strain JM109. (TIF) [file pone.0033342.s009.tif]

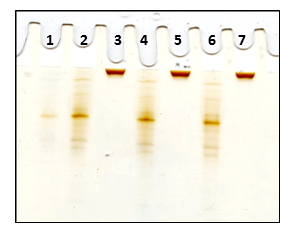

Supplement: Figure S10 — Separation of EutC1–19-EGFP from broken and intact Eut shells by native polyacrylamide electrophoresis. Visualization of protein migration by silver stain of native gel. EGFP control is shown in lane 1, followed by broken (lane 2) and intact (lane 3) Eut BMCs from S. enterica cells harboring EutC1–19-EGFP; broken (lane 4) and intact (lane 5) recombinant EutSMNLK BMCs co-expressing EutC1–19-EGFP; and broken (lane 6) and intact (lane 7) recombinant EutS BMCs from E. coli C2566 cells co-expressing EutC1–19-EGFP. (TIF) [file pone.0033342.s010.tif]
