# Supplementary material for: Engineered Protein Nano-Compartments for Targeted Enzyme Localization
Source: PLoS One. 2012 Mar 12;7(3):e33342. doi: 10.1371/journal.pone.0033342 (PMC3299773; doi:10.1371/journal.pone.0033342)
Supplement: Table S1 — Quantification of the distribution of recombinant Eut shells in E. coli . Thin cell sections of E. coli expressing EutS or EutSMNLK were observed by TEM. Assuming an average E. coli cell has a height of 2 µm and a diameter of 0.5 µm, about 20 thin sections (90 nm in width) perpendicular to the axis can be cut from each cell. An average recombinant Eut protein shell has a diameter of 100–200 nm. The average E. coli cell would have about 20 cross-sections parallel to the circular base, of which only two would pass through an engineered Eut shell. Even if 100% of E. coli had a recombinant Eut compartment, the actual fraction of cell cross-sections showing the phenotype would be around 10%. For sections parallel to the height of the cell, less than 40% would be expected to display the shell, and the number showing compartments at close to their maximum width will be even lower. (DOC) [file pone.0033342.s012.doc]

**Table S1.** **Quantification of the distribution of recombinant Eut shells in *E. coli*.**

| ***E. coli* strain** | **Gene combination** | **Total number of cells** | **Number of *E. coli* thin sections with engineered shells** | **% of E. coli thin sections with engineered shells** |
| --- | --- | --- | --- | --- |
| JM109 | EutS | 400 | 63 | 15.75 |
| JM109 | EutSMNLK | 270 | 60 | 22.22 |

Thin cell sections of *E. coli* expressing EutS or EutSMNLK were observed by TEM. Assuming an average *E. coli* cell has a height of 2 µm and a diameter of 0.5 µm, about 20 thin sections (90 nm in width) perpendicular to the axis can be cut from each cell. An average recombinant Eut protein shell has a diameter of 100-200 nm. The average *E. coli* cell would have about 20 cross-sections parallel to the circular base, of which only two would pass through an engineered Eut shell. Even if 100% of *E. coli* had a recombinant Eut compartment, the actual fraction of cell cross-sections showing the phenotype would be around 10%. For sections parallel to the height of the cell, less than 40% would be expected to display the shell, and the number showing compartments at close to their maximum width will be even lower.
