# Supplementary material for: Engineered Protein Nano-Compartments for Targeted Enzyme Localization
Source: PLoS One. 2012 Mar 12;7(3):e33342. doi: 10.1371/journal.pone.0033342 (PMC3299773; doi:10.1371/journal.pone.0033342)
Supplement: Table S2 — Quantification of the distribution of engineered Eut protein shells in E. coli by fluorescence microscopy. (DOC) [file pone.0033342.s013.doc]

**Table S2. Quantification of the distribution of engineered Eut protein shells in *E. coli* by fluorescence microscopy.**

| ***E. coli* strain** | **Gene combination** | **Total number of cells** | **Number of cells with green fluorescent foci** | **% of cells with green fluorescent foci** | **Average number of green fluorescent foci per cell** |
| --- | --- | --- | --- | --- | --- |
| C2566 | EGFP | 867 | 0 | 0 | 0 |
| C2566 | EutC^1-19^-EGFP | 881 | 4 | 0.4 | 0 |
| C2566 | EGFP + EutS | 876 | 4 | 0.5 | 0 |
| C2566 | EutC^1-19^-EGFP + EutS | 1023 | 890 | 87 | 1.2 |
| C2566 | EGFP + EutMNLK | 834 | 1 | 0.1 | 0 |
| C2566 | EutC^1-19^-EGFP + EutMNLK | 964 | 6 | 0.6 | 0 |
| C2566 | EGFP + EutSMNLK | 843 | 2 | 0.2 | 0 |
| C2566 | EutC^1-19^-EGFP + EutSMNLK | 912 | 766 | 84 | 1.3 |
| C2566 | EGFP + EutS-G39V | 813 | 2 | 0.3 | 0 |
| C2566 | EutC^1-19^-EGFP + EutS-G39V | 861 | 4 | 0.4 | 0 |
| JM109 | EGFP | 992 | 7 | 0.7 | 0 |
| JM109 | EutC^1-19^-EGFP | 897 | 7 | 0.8 | 0 |
| JM109 | EGFP + EutS | 901 | 5 | 0.6 | 0 |
| JM109 | EutC^1-19^-EGFP + EutS | 986 | 828 | 84 | 1.2 |
| JM109 | EGFP + EutMNLK | 931 | 7 | 0.8 | 0 |
| JM109 | EutC^1-19^-EGFP + EutMNLK | 839 | 5 | 0.6 | 0 |
| JM109 | EGFP + EutSMNLK | 941 | 17 | 1.8 | 0 |
| JM109 | EutC^1-19^-EGFP + EutSMNLK | 1011 | 799 | 79 | 1.1 |
| JM109 | EGFP + EutS-G39V | 821 | 24 | 2.9 | 0 |
| JM109 | EutC^1-19^-EGFP + EutS-G39V | 886 | 71 | 8 | 0 |
