# Supplementary material for: Engineered Protein Nano-Compartments for Targeted Enzyme Localization
Source: PLoS One. 2012 Mar 12;7(3):e33342. doi: 10.1371/journal.pone.0033342 (PMC3299773; doi:10.1371/journal.pone.0033342)
Supplement: Methods S1 — Supporting Methods. (DOC) [file pone.0033342.s014.doc]

**SUPPORTING INFORMATION**

**Engineered protein nano-compartments for targeted enzyme localization**

Swati Choudhary^1^, Maureen B. Quin^1^, Mark A. Sanders^2^, Ethan T. Johnson^1^ and Claudia Schmidt-Dannert^1,*^

**1** Department of Biochemistry, Molecular Biology and Biophysics, University of Minnesota, St. Paul, Minnesota, USA, **2** University Imaging Centers, University of Minnesota, St. Paul, Minnesota, USA

^*^ E-mail: schmi232@umn.edu

**I. Supporting Methods**

**Microbiological methods.** *E. coli* strains JM109 and the BL21 derivative C2566 were obtained from Sigma and New England Biolabs, respectively. *Salmonella enterica* serovar Typhimurium LT2 was a kind gift from Dr. Jeffrey A. Gralnick (University of Minnesota).

**General Molecular Biology Methods. Illustra GFX PCR DNA and gel band purification kit (GE Healthcare) was used for all agarose gel and PCR purifications.** Plasmid DNA was purified using Promega Wizard *Plus* SV Minipreps DNA Purification kit according to the manufacturer’s instructions. **Restriction enzymes and T4 DNA ligase were obtained from New England Biolabs and Invitrogen, respectively. Standard protocols were used for restriction digests and DNA ligation .**

**BioBrick^TM^-based expression vectors. The BioBrick^TM^ strategy allows for rapid sub-cloning of genes into several vectors using the same set of restriction enzymes (Fig. S1). Our in-house BioBrick^TM^ expression vectors have a constitutively active modified lac promoter (P_lac*_) .** Various open reading frames can be inserted between the BglII and NotI sites downstream of **P_lac*_**. pUCBB (ampicillin resistance) and pACBB (chloramphenicol resistance) have ColE1 and p15A origins of replication, respectively, while pBBRBB (kanamycin resistance) is derived from the broad-host-range vector pBBR1MCS .

**Cloning of Eut genes.** Eut shell genes were amplified from *Salmonella enterica* LT2 genomic DNA (ATCC Catalog no. **700720D-5) using gene-specific primers. Restriction sites for BglII and NotI were added to the 5’ end of the forward and reverse primers, respectively. Each PCR product was gel purified and digested with BglII and NotI, followed by ligation with BglII/NotI digested pUCBB. All cloned sequences were confirmed by DNA sequencing.**

EutS, EutMN and EutLK were cloned into our in-house BioBrick^TM^ constitutive expression vector pUCBB (**Fig. S1**). Their expression cassettes were stacked sequentially to produce the vectors pUCBBEutMNLK and pUCBBEutSMNLK. **To stack the Eut expression cassettes, pUCBBEutMN and pUCBBEutLK were digested with EcoRI/SpeI and EcoRI/XbaI respectively. XbaI and SpeI produce compatible DNA ends that upon ligation generate a ‘scar’ which cannot be recognized by either enzyme. The P_lac*_-EutMN DNA fragment was ligated into digested pUCBBEutLK to produce the vector pUCBBEutMNLK. Next, pUCBBEutS was digested with EcoRI/SpeI, and the P_lac*_-EutS fragment ligated into EcoRI/XbaI digested pUCBBEutMNLK to produce the final vector pUCBBEutSMNLK.** EutM, EutN, EutL and EutK were also cloned singly to study their individual contributions to BMC formation. For expression in *S. enterica*, EGFP, EutC^1-19^-EGFP and EutG^1-19^-EGFP were further sub-cloned into our in-house broad-host-range BioBrick^TM^ expression vector pBBRBB.

**Protein analysis.** *E. coli* C2566 cells were grown at 37 °C for SDS/PAGE analysis of recombinant Eut protein expression, which was performed using standard methods . Total cellular protein was extracted from pelleted overnight cultures using BugBuster (Novagen) extraction reagent. The extracts were centrifuged (12,000 rpm, 15 minutes, 4 °C) to separate the soluble and insoluble fractions. Protein concentrations were determined using Bio-Rad protein assay reagent (Bio-Rad). For studying expression of recombinant Eut proteins, PAGE was performed using 15% SDS gels and Bio-Rad Mini-Protean II electrophoresis cells as per the manufacturer’s instructions. The protein gels were subsequently stained with Bio-Safe Coomassie Blue (Bio-Rad). Silver staining was used to visualize purified Eut BMC proteins.

**Western detection of EutC^1-19^-EGFP from purified BMCs.** Purified native Eut BMCs and recombinant EutSMNLK and EutS BMCs harboring EutC^1-19^-EGFP were broken by sonication. 10 µg of broken and intact BMCs were loaded in separate lanes of a 10 % native polyacrylamide gel. Following electrophoresis under non-denaturing conditions, migration of proteins was visualized by silver staining. Proteins were also transferred to a PVDF membrane (Roche), and GFP was detected using a primary anti-GFP antibody and a secondary horseradish peroxidase- conjugated antibody. A chromogenic developing solution was prepared using a one:one ratio of luminol/enhancer solution and stable peroxide solution (Thermo Scientific), and was applied to the membrane. The membrane was placed in a film cassette and was exposed to film (Bioexpress) for 2 minutes, and the film was subsequently developed.

**Anti-GFP immunofluorescence studies.** *E. coli* cells were suspended in PBS for microwave-assisted low temperature processing. Microwave-assisted low temperature processing was conducted in a Pella Biowave Pro microwave processor equipped with a ColdSpot™ load cooler, vacuum system, and variable wattage. Bacterial pellets were initially fixed using 0.1 % glutaraldehyde, 4 % paraformaldehyde, and 0.1 M sodium phosphate buffer (pH 7.2), in the microwave processor at 150 watts for 12 min (5 min on, 2 min off, 5 min on) at 4 °C. Subsequently, the cells were mounted on 10-welled microscope slides, permeabilized in -20 °C methanol for 10 min, and allowed to air dry. Drops of blocking buffer (PBS (pH 7.2) with 5 % normal goat serum, 1 % glycerol, 0.1 % BSA, 1 % fish skin gelatin, and 0.04 % sodium azide) were placed on the attached cells for 15 minutes at room temperature. Specimens were reacted overnight at 4 ^o^C with the anti-GFP antibody diluted 1:50 (Invitrogen, Catalog no. A11122) and then incubated with Alexa 568 goat anti-rabbit IgG for 2 h at 37 °C. Finally, the cells were washed and mounted in Prolong Gold with DAPI. Preparations were viewed using the E800 microscope as described in the main text.

**Anti-GFP immunogold labeling and TEM.** After microwave processing, the cell pellets were pre-embedded in 2 % NuSieve agarose (Cambrex Life Sciences), and washed twice for 30 min each in phosphate buffer at 4 °C. Dehydration substitution procedures were conducted in a block of dry ice with 1.5 ml microfuge tubes placed in 100 % solvent filled pre-drilled holes. Samples were dehydrated/substituted in 50 %, 75 % and 96 % Methanol, 1:1 96% Methanol: LR White resin, 100% LR White sequentially at dry ice temperatures in the presence of microwaves with the following wattages and times: 150 watts for 12 min (5 min on, 2 min off, 5 min on) each. The LR White infiltrated samples where then embedded with fresh LR White resin followed by polymerization at 42 °C for 18 h. The polymerized blocks were sectioned (90 nm) with diamond knives and placed on formvar-coated nickel 200 mesh grids.

For immunogold labeling, sections were labeled for anti-GFP (Invitrogen, Catalog no. A11122) using the indirect immunogold labeling technique. Grids with sections were floated on drops of blocking buffer, consisting of PBS, pH 7.2, with 5 % normal goat serum, 1 % glycerol, 0.1 % bovine serum albumin (Fraction V; Sigma), 1 % fish skin gelatin, and 0.04 % sodium azide for 15 minutes at room temperature (RT). Specimens were reacted overnight at 4 ^o^C with the anti GFP antibody diluted 1:50. After washing seven times in droplets of PBS, the sections were incubated with 20 nm goat anti-rabbit IgG (1:50 dilution, GE Healthcare). After washing seven times with droplets of PBS, the sections were fixed in 1 % glutaraldehyde followed by rinsing on a droplet of water eight times. All sections were stained for 5 minutes with uranyl acetate and 5 minutes with lead citrate before observation on a Phillips CM 12 TEM.

**Nile Red assay:** Overnight cultures of bacteria were incubated with Nile Red (final concentration 1 µg/ml) for five minutes in the dark. Fluorescence visualization was performed using the Nikon E800 microscope as described in the main text.

**References**

1. Sambrook J, Fritsch EF, Maniatis T (1989) Molecular cloning: a laboratory manual: Cold Spring Harbor Lab Press, Cold Spring Harbor, NY.

2. Schmidt-Dannert C, Umeno D, Arnold FH (2000) Molecular breeding of carotenoid biosynthetic pathways. Nat Biotechnol 18: 750-753.

3. Vick JE, Johnson ET, Choudhary S, Bloch SE, Lopez-Gallego F, et al. (2011) Optimized compatible set of BioBrick vectors for metabolic pathway engineering. Appl Microbiol Biotechnol 92: 1275-1286.

4. Kovach ME, Phillips RW, Elzer PH, Roop RM, Peterson KM (1994) pBBR1MCS: a broad-host-range cloning vector. Biotechniques 16: 800-802.

**II. Supporting Figure Legends**

**Figure S1.** **BioBrick^TM^ vectors and strategy for stacking multiple genes into a single plasmid.** **(A)** Our in-house BioBrick^TM^ vectors contain an expression cassette with a constitutive promoter (P_lac*_) and an EGFP reporter. **(B)** Cloning of Eut BMC shell genes into pUCBB. (i) EutS, EutMN and EutLK were cloned downstream of the constitutive P_lac*_ promoter (blue arrow) using BglII and NotI. (ii) and (iii) E**xpression cassettes for EutMNLK** and **EutSMNLK** were created as described in **Supporting Methods**.

**Figure S2.** **SDS/PAGE analysis showing recombinant expression of *S. enterica* Eut shell proteins in *E. coli*.** **(A)** Overexpression of Eut shell proteins in the *E. coli* strain C2566. **(B)** Overexpression of Eut shell proteins in the *E. coli* strain JM109. **(c)** Overexpression of wild type EutS and the EutS-G39V mutant in *E. coli* strains C2566 and JM109. 15µg soluble protein fraction was loaded in each lane. Expected protein sizes are as follows: EutS (11.6kDa), EutM (9.8 kDa), EutN (10.4kDa), EutL (22.7 kDa), EutK (17.5 kDa), and EGFP (26.9 kDa). Proteins were stained with Coomassie Blue.

**Figure S3.** **Transmission electron micrographs of thin sections of recombinant *E. coli* expressing *S. enterica* Eut shell proteins.** **(A-C)** *E. coli* expressing recombinant EutS contain properly delimited shells (*E. coli* strain used in **A**: C2566, and in **B**, **C**: JM109). **(D-F)** *E. coli* expressing recombinant EutM form thick axial filaments that interfere with separation after cell-division (*E. coli* strain used in **D, E**: C2566, and in **F**: JM109). **(G)** *E. coli* JM109 expressing recombinant EutN. **(H)** *E. coli* JM109 expressing recombinant EutL. **(I)** *E. coli* JM109 expressing recombinant EutK shows an electron translucent region in the middle of the cell. **(J)** An electron dense region is visible in *E. coli* JM109 co-expressing recombinant EutM and EutN. **(K)** Intracellular filaments are formed in *E. coli* JM109 co-expressing recombinant EutL and EutK. **(L-N)** Clearly defined shells are observed in *E. coli* JM109 expressing recombinant EutSMNLK. **(O-Q)** Co-expression of EutSMNLK and EutC^1-19^-EGFP results in the formation of compartments that are morphologically similar to the shells observed *in vivo* by expression of either EutS or EutSMNLK alone. (*E. coli* strain used in **O**: C2566, and in **P, Q**: JM109). Arrows indicate the location of recombinant shells. (Scale bar: 200nm).

**Figure S4.** **Localization of EutC^1-19^-EGFP in recombinant *E. coli* JM109 cells expressing *S. enterica* Eut shell proteins.** Fluorescence microscopy images of *E. coli* JM109 cells co-expressing EGFP or EutC^1-19^-EGFP with EutS (wild type and the G39V mutant), EutMNLK or EutSMNLK. See **Table S2** for the quantification of EGFP localization in recombinant *E. coli*, and **Fig. 4** for the localization of EutC^1-19^-EGFP in the *E. coli* C2566 strain. Cell boundaries are shown by the DIC images.

**Figure S5.** **Localization of EutC^1-19^-EGFP in recombinant *E. coli* C2566 cells expressing various combinations of *S. enterica* Eut shell proteins.** Fluorescence microscopy images of *E. coli* C2566 cells with constructs for constitutive expression of EGFP or EutC^1-19^-EGFP with EutM, EutN, EutL, EutK, EutMN and EutLK. In the absence of EutS, there is no discrete fluorescent localization of EutC^1-19^-EGFP, which indicates that EutS is required for targeting EutC^1-19^-EGFP to the engineered microcompartments. Cell boundaries are shown by the DIC images.

**Figure S6. Nile Red staining of recombinant *E. coli* expressing EutC^1-19^-EGFP.** *E. coli* C2566 cells co-expressing EutC^1-19^-EGFP and EutS or EutSMNLK were stained with the fluorescent, lipophilic inclusion body stain Nile Red. Co-localization of red and green fluorescence was not observed, indicating that the recombinant Eut shells are not inclusion bodies nor are the surrounded by a hydrophobic matrix.

**Figure S7. Nile Red staining of recombinant *E. coli* expressing NSC1.** *E. coli* C2566 cells co-expressing the cyanobacterial carotenoid cleavage dioxygenase NSC1 either alone or with EutC^1-19^-EGFP. While red fluorescent puncta corresponding to inclusion bodies were observed in the presence of NSC1, co-localization of red and green fluorescence was not seen, showing that EutC^1-19^-EGFP is not targeted to NSC1 inclusion bodies.

**Figure S8. Transmission electron micrographs of partially purified protein compartments. (A)** Native Pdu BMCs isolated from *S. enterica*. **(B)** Native Eut BMCs and recombinant Eut protein shells isolated from cells not expressing the cargo protein EutC^1-19^-EGFP. From left to right: Native Eut BMCs isolated from *S. enterica*, recombinant EutSMNLK shells isolated from *E. coli* C2566, and recombinant EutS shells isolated from *E. coli* C2566. Scale bar: 100nm.

**Figure S9. Immunofluorescence analysis of EutC^1-19^-EGFP localization in recombinant *E. coli* expressing Eut shell proteins.** EGFP, anti-GFP antibody (red) and merged EGFP-anti-GFP antibody fluorescence signals from *E. coli* cells with constructs for constitutive expression of EGFP or EutC^1-19^-EGFP with EutS or EutSMNLK. **(A)** anti-GFP immunofluorescence studies in the *E. coli* strain C2566. **(B)** anti-GFP immunofluorescence studies in the *E. coli* strain JM109.

**Figure S10. Separation of EutC^1-19^-EGFP from broken and intact Eut shells by native polyacrylamide electrophoresis.** Visualization of protein migration by silver stain of native gel. EGFP control is shown in lane 1, followed by broken (lane 2) and intact (lane 3) Eut BMCs from *S. enterica* cells harboring EutC^1-19^-EGFP; broken (lane 4) and intact (lane 5) recombinant EutSMNLK BMCs co-expressing EutC^1-19^-EGFP; and broken (lane 6) and intact (lane 7) recombinant EutS BMCs from *E. coli* C2566 cells co-expressing EutC^1-19^-EGFP.

**Video S1: Dynamics of EutC^1-19^-EGFP in *S. enterica* grown on ethanolamine.**

Representative time-lapse movie of *S. enterica* cells harboring pBBRBB-EutC^1-19^-EGFP, and grown in the presence of ethanolamine. Discrete fluorescent foci are observed to be in motion within the *S. enterica* cells, suggesting that Eut BMCs (which would be expected to encapsulate EutC^1-19^-EGFP) are moving around within the cell. Time stamp on video indicates elapsed time. Preparations were viewed using a Nikon Eclipse E800 photomicroscope.  Time-lapse images were collected at 15 second intervals. Shutters were opened only during camera exposure.
